# Euclidean Graphs as Crack Pattern Descriptors for Automated Crack Analysis in Digital Images

**DOI:** 10.3390/s22165942

**Published:** 2022-08-09

**Authors:** Alberto Strini, Luca Schiavi

**Affiliations:** Istituto per le Tecnologie della Costruzione, Consiglio Nazionale delle Ricerche (ITC-CNR), Via Lombardia 49, I-20098 San Giuliano Milanese, MI, Italy

**Keywords:** line-shaped feature description, crack path descriptor, image segmentation, computer vision, graph algorithms, Dijkstra’s algorithm, performance evaluation indicators, cracked cement surface, structural damage assessment, autonomous structure inspection

## Abstract

Typical crack detection processes in digital images produce a binary-segmented image that constitutes the basis for all of the following analyses. Binary images are, however, an unsatisfactory data format for advanced crack analysis algorithms due to their sparse nature and lack of significant data structuring. Therefore, this work instead proposes a new approach based on Euclidean graphs as functional crack pattern descriptors for all post-detection analyses. Conveying both geometrical and topological information in an integrated representation, Euclidean graphs are an ideal structure for efficient crack path description, as they precisely locate the cracks on the original image and capture salient crack skeleton features. Several Euclidean graph-based algorithms for autonomous crack refining, correlation and analysis are described, with significant advantages in both their capabilities and implementation convenience over the traditional, binary image-based approach. Moreover, Euclidean graphs allow the autonomous selection of specific cracks or crack parts based on objective criteria. Well-known performance metrics, namely precision, recall, intersection over union and F1-score, have been adapted for use with Euclidean graphs. The automated generation of Euclidean graphs from binary-segmented images is also reported, enabling the application of this technique to most existing detection methods (e.g., threshold-based or neural network-based) for cracks and other curvilinear features in digital images.

## 1. Introduction

Surface crack monitoring is of primary importance in the structural assessment of structures made from concrete [1,2] or other materials [3,4], and the identification and analysis of surface cracks is therefore gaining increasing interest both in the field for structural diagnostics [5,6] and in the laboratory for the study of material properties [7,8]. In particular, the demand for efficient and automated crack detection and analysis methods for structural diagnostics in building structures will undoubtedly increase in the forthcoming years because of the progressive ageing of civil constructions, especially in the Western world. As an example, according to EU sources [9], in several European countries more than 50% of the residential building stock was built before 1969 and more than 25% was built before 1945 (corresponding to ages of more than 50 and 75 years in 2021, respectively). Moreover, premature failure can also be a problem even for relatively recent and critical structures such as bridges [2]. Traditional visual inspection is currently being progressively substituted by image-based approaches [2,10] because of the great potential of the related computational analysis and the objectiveness of the resulting documentation. In addition, the development of advanced algorithms prospectively enables the goal of a fully automated crack detection and analysis process. Among the different techniques studied for the detection of cracks and other defects in the field and in the laboratory (e.g., digital image correlation [11,12], stereoscopic image analysis [13] and laser scanning [3,14,15,16]), the acquisition and analysis of digital crack images [7,8,17,18,19,20] is the most common approach, thanks to the relatively simple and inexpensive acquisition equipment used. Furthermore, the recent availability of affordable robot-mounted camera systems, both wheeled and airborne (drones), has paved the way for a growing effort in the development of remote monitoring platforms with great potential in the damage assessment of buildings, bridges and other structures that are difficult to reach by direct inspection [21,22,23,24,25,26]. In this framework, the development of computer systems for the automated detection, selection and analysis of cracks in digital images appears as an essential tool to unleash the full potential of these technologies.

The analysis of a crack image is typically subdivided into a first detection phase, where the possible surface cracks are identified through a specific image segmentation process, and a subsequent post-detection phase, where the recognized cracks are refined, selected and analyzed. Several methods were studied for automatic and semiautomatic crack detection, from traditional image processing techniques such as threshold segmentation or edge detection [23,27,28], to more advanced strategies based on convolutional neural networks (CNNs), deep learning or hybrid approaches [2,6,25,26,29,30,31,32]. Nevertheless, most crack detection algorithms result in a binary image where pixels putatively related to cracks are identified and marked against the background [17,18,25,27,28,32]. Currently, the obtained binary segmented image is typically refined with morphological algorithms (e.g., to filter-out noise and improve the crack representation [33,34,35]) and then used as a basis for all the following crack analyses [4,7,36].

Binary images, however, are not the best data format for the implementation of advanced crack refining and analysis algorithms, due to their very sparse nature and the lack of any meaningful data structuring (aside from the Cartesian coordinates of each identified pixel). This results in the necessity to identify each needed crack feature by direct search on the binary image itself [20]. Moreover, the direct use of binary segmented images for crack analysis inevitably poses strict requirements for the crack detection process and the successive refining algorithms in order to avoid the introduction of measurement errors (e.g., in the determination of crack width [27]).

An ideal data structure for crack description should instead capture the essentials of the crack pattern, ensuring an effective computational availability of the main crack path features for direct analysis (e.g., crack end-points and bifurcations). At the same time, such structure should provide for a quick and precise localization of the crack path and the related features on the original grayscale image when this is required for specific measurements.

**Figure 1:**
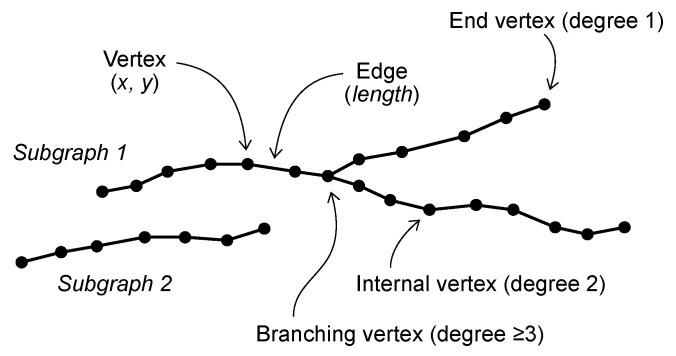
Euclidean graph composed by two disconnected subgraphs. Each vertex is associated with its spatial coordinates in the Euclidean plane. Each edge is associated with a weight proportional to the Euclidean distance between its vertices.

A weighted graph is a set of vertices (nodes) connected by edges (links), each of the latter having a numerical value (weight) associated with it. An Euclidean graph (EG) is a weighted graph where each vertex corresponds to a point in an Euclidean space (in this case, the Euclidean plane, Figure 1) and each edge weight is proportional to the Euclidean distance between its two endpoints [37]. As ordinary graphs, EGs can consist of several subgraphs (i.e., graph components that are internally connected by edges but mutually disconnected). Each EG vertex can also be classified based on its degree (i.e., the number of connected edges) as an isolated vertex (degree 0), end vertex (degree 1), internal vertex (degree 2) or branching vertex (degree ≥ 3).

EGs appear as ideal structures for the functional description of the crack pattern, as they precisely identify the crack path on the original image, thus enabling at the same time a quick localization on the image itself and an efficient capture of salient crack skeleton features, both topological and geometrical (e.g., branches, end points, cycles, length and form factor). Most crack analyses, such as length [23], tortuosity [7], orientation [35] and fractal dimension [38,39,40], can therefore be performed directly on the EG itself, taking full advantage of its rich information content without resorting to the segmented image. EG representation appears also as an optimal starting point for more sophisticated analyses such as the automated crack pattern recognition for structural assessment [41]. Specific crack analyses such as the measurement of crack width can instead be carried out on the original grayscale image using the EG representation as a guide for crack localization and for the automated selection of the crack parts to be measured, thus avoiding any issues related to image manipulation during the identification process. Moreover, different EGs can be quantitatively compared according to their overlapping degree, enabling the use of well-known performance metrics, such as intersection over union and F1-scores. On top of that, the EG-based description of the crack path provides a very convenient basis for the generation of machine-readable documentation for archiving and later analysis.

In this work, it is therefore proposed to use an EG-based description of the crack pattern for all of the post-detection refinement and analysis processes instead of using the binary-segmented image. The EG is obtained from the latter through a skeletonization step that reduces all of the detected cracks to their single-pixel-wide medial line.

This approach offers, among the others, the following advantages:

- The skeletonized crack image is explored only once during the EG generation, then all of the related information is recorded in the EG, ensuring a very wide computational availability for following processes.
- The topological and geometrical information conveyed by EGs allows the implementation of tailored algorithms for the refinement of the detected cracks and for their selection, classification, processing and analysis.
- Particular cracks and crack zones can be algorithmically identified and classified on the EG itself according to specific requirements (e.g., avoidance of crack ends and bifurcations during crack width measurements).
- The position information associated with each EG vertex allows a precise crack localization on the original image for specific measurement tasks (e.g., accurate crack width determination).
- The EG can constitute the foundation of a machine-readable, high-resolution crack pattern documentation for archiving and later analysis (e.g., image-to-image crack correlation).

**Figure 2:**
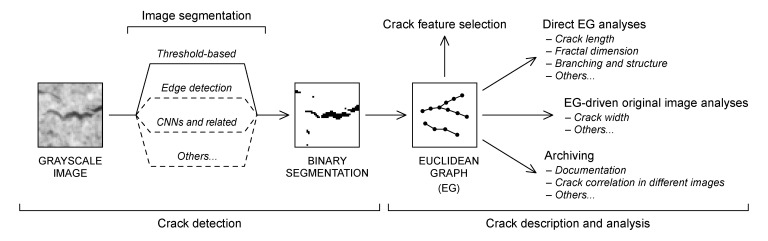
EG-mediated image analysis process. The EG description of the crack pattern can be derived from any segmentation algorithm that results in a binary image, allowing the implementation of automated, EG-based crack analyses to most of the existing crack detection methods.

Furthermore, EGs allow for easy manipulation for the refinement of a crack pattern, such as edge insertion and deletion for crack merging and separation. EGs can also be modified for smoothing (i.e., changing the vertex position to provide a less jagged path as illustrated in Section 3.4.3) or simplified to derive a more compact representation (e.g., for algorithm optimization, see Section 3.3.5). EGs thus constitute an ideal interface between crack detection and crack analyses processes, allowing the full exploitation of the information provided from the former with efficient and versatile algorithms. The whole image analysis process is thus subdivided (Figure 2) into a first phase, where a binary segmentation is derived from the original grayscale image using a suitable crack detection process, and a second phase, where a functional EG description of the crack pattern is derived from the binary segmentation in order to constitute the basis for the following analyses. The latter can then be carried out both directly on the EG itself and on the original grayscale image using the EG as a guide.

In the present work, the potential of EGs as a crack pattern descriptor is illustrated by means of a simple yet comprehensive analysis process, specifically developed for demonstration purposes. This process aims at the analysis of crack images captured during the tensile strength test of reinforced cementitious renders used as a base coat for building thermal insulation systems. The typical procedure for this laboratory test requires the manual identification and evaluation of cracks in a sequential image series acquired from a sample under increasing unidirectional tensile strain. This is a particularly challenging task for automated image analysis, because thin cracks must be identified and measured on a rough surface, which causes a significant texture in the resulting image, imposing severe requirements both on the image acquisition system and on the analysis process. Moreover, the cracks identified in the whole image sequence set must be correlated in order to reconstruct the crack evolution during the test. In the present work, the crack detection is implemented with a simple threshold-based binary segmentation process. The resulting binary image is then skeletonized in order to derive a raw, primal EG that is further processed to provide a refined, final EG. The advantages of the EG-based crack description are apparent even in the early processing of the primal EG and are highlighted by illustrating an implementation that includes noise deletion, subgraph merging and artefact removal. The reported process, albeit a relatively simple example, allows the generation of a fairly good final EG that describes the crack pattern in the image and constitutes the basis for the following analyses. The quality of the obtained final EG is assessed by using an EG-based reference ground truth with traditional metrics specifically adapted to the comparison of EGs. Examples of crack analyses carried out directly on the final EG (crack length and crack branching) and on the original grayscale image by direction of crack EG (crack width) are described alongside an image-to-image crack correlation analysis in the whole time-series image set. The advantages obtained in the refinement of the crack paths and in the following crack analyses are discussed and compared with the traditional implementation based on the direct analysis of the binary segmented image.

It is worth noting that the EG representation of a crack network can be handled with a wide variety of methods thanks to its compact description of both topological and geometrical crack skeleton features, allowing the development of a broad family of procedures for accurate crack pattern refining and analysis. Some of these are described in the present work, but this is only a small set of possible processing strategies made available by this approach, which allows to merge the large collection of algorithms developed for graph processing with the extensive variety of digital image analysis methods reported in the literature.

Moreover, an EG representation of a curvilinear feature can be easily derived from any binary-segmented image. EG-mediated analyses can therefore be applied to any detection process that results in a binary segmentation, such as threshold-based methods or pixel-wise CNN-based algorithms.

The implementation of an EG-based pattern description can therefore provide great advantages in the image processing of cracks and other line-shaped features, leading to a unified approach for all post-detection refinements and analyses and allowing the full exploitation of the potentials given by most of the classic and advanced detection methods.

## 2. Experimental Methods

The application of EGs in the analysis of crack images is illustrated in the present work by studying the cracks developed during the tensile strength test of reinforced cementitious renders used as a base coat for building thermal insulation systems. The cracks were generated by applying a controlled strain on a 600 × 100 mm strip sample using a static uniaxial tensile testing machine, generating a sequence of up to five images recorded at an increasing strain level.

### 2.1. Image Acquisition

All images were recorded with a 6576 × 4384 pixel CCD monochromatic camera (Prosilica GX 6600, Allied Vision Technologies GmbH, Stadtroda, Germany) equipped with a 50 mm, *f*/2 lens (Makro Planar T* 2/50, Zeiss). The illumination source used to optimize the crack detection was a 180 mm diffused side annular red LED light (FPR-180, CCS Inc., Kyoto, Japan). A lens-mounted 660 nm passband filter centered on LED emission band (BP660, Midwest Optical Systems Inc., Palatine, IL, USA) ensured negligible disturbance by ambient light.

The acquisition system was installed on a tensile testing machine with a geometry optimized in order to image a 10 × 10 cm sample surface with maximizing resolution. The resulting sample-image sensor distance was 344 mm, with 23.8 μm/px nominal reproduction scale at the sample surface. The actual reproduction scale can vary slightly in each setup because of sample positioning tolerances on the testing machine and must be evaluated each time by means of a calibrated target. The illumination angle was chosen as a compromise between the need to maximize crack contrast (that requires a large angle of incidence) and to minimize the shading due to the sample surface roughness (that requires a small angle of incidence instead). In this work, the annular illumination system was positioned at a 50 mm distance from the sample surface.

All images are recorded at medium lens aperture (*f*/8) in order to optimize resolution and depth of field. Images were recorded as grayscale, uncompressed files (.bmp format, no gamma correction) with 8 bits per pixel using the manufacturer’s acquisition software (AVT UniCam Viewer 1.2, Allied Vision). Illumination source power and exposure time were adjusted in order to optimize the image intensity histogram.

### 2.2. Reference Ground Truth

An EG-based reference ground truth for the sample described in this work was obtained by means of the manual examination of the original image used for the automated analysis process. The computer-assisted visual inspection allowed to identify even the finest cracks (width ≤ 1 px) that were recorded on the image as a low-contrast, 1-pixel-wide feature. The actual EG representation was generated by software, tracking the crack path between closely spaced points visually identified on a high-magnification representation of the sample image.

### 2.3. Software

The software used for EG-based crack image analysis was developed in-house in C language using the Xcode 9.2 (LLVM/Clang) development system (Apple Inc., Cupertino, CA, USA). Camera image files (.bmp file format) were read with custom routines and stored internally as an 8-bit grayscale representation. Images for illustrations were saved as PDF or PNG file using operating system routines (Cocoa, Apple). Figures and illustrations were created with Affinity Designer 1.10 (Serif Europe Ltd., Nottingham, UK).

### 2.4. EG-Mediated Image Processing

**Figure 3:**
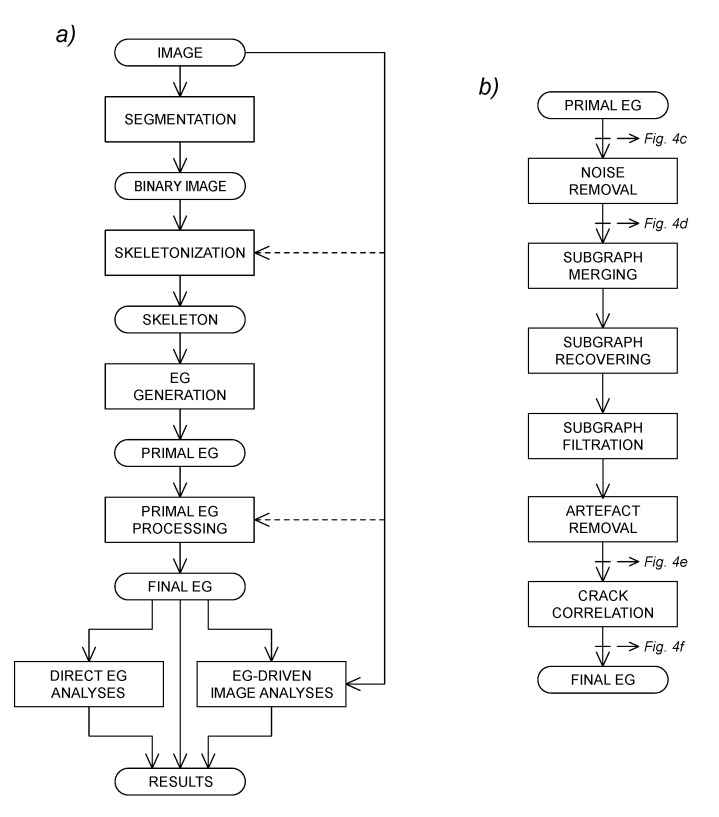
EG-mediated image processing flowchart. Complete image processing with primal EG generation and finishing to obtain the refined final EG suitable for downstream analyses. Advanced algorithms for skeletonization and processing of primal EG can also take advantage of the direct access to original image data (**a**). Detailed finishing process of primal EG (**b**). The outcome of selected processing steps is shown in Figure 4.

The whole analysis process is depicted in the flowchart of Figure 3a. The image segmentation step is responsible for the primary identification of fractures, resulting in a binary image that categorizes pixels as putatively belonging to fractures (foreground) or to the sample surface (background).

The binary segmented image is then skeletonized (i.e., all pixel clusters are reduced to their centerline). A raw, primal EG is directly derived from the skeletonized image with complete information retention. The primal EG is then processed in order to obtain the final EG, which is a functional description of the crack pattern captured in the original image. This latter step is depicted in detail in Figure 3b. In the described implementation, a strictly sequential process computes the final EG, but tailored algorithms can also take advantage of direct access to the original image even in the intermediate phases, particularly in the skeletonization and EG processing steps (dashed arrows in Figure 3a). Subsequent crack analyses can then be performed either directly on the final EG itself or on the original image using the final EG as a guide. The details of image processing, EG generation and crack analysis methods are described in Section 3 and in the Supplementary Information.

## 3. Results and Discussion

The potential of EG-based analysis will be illustrated using a simple automated crack image analysis process as example. The reported process is based on a threshold binarization detection that results in a relatively low-quality binary segmentation, which is ideal for demonstrating various EG-based refinement techniques. The finished crack description will then be used to demonstrate several EG-based fracture analyses.

**Figure 4:**
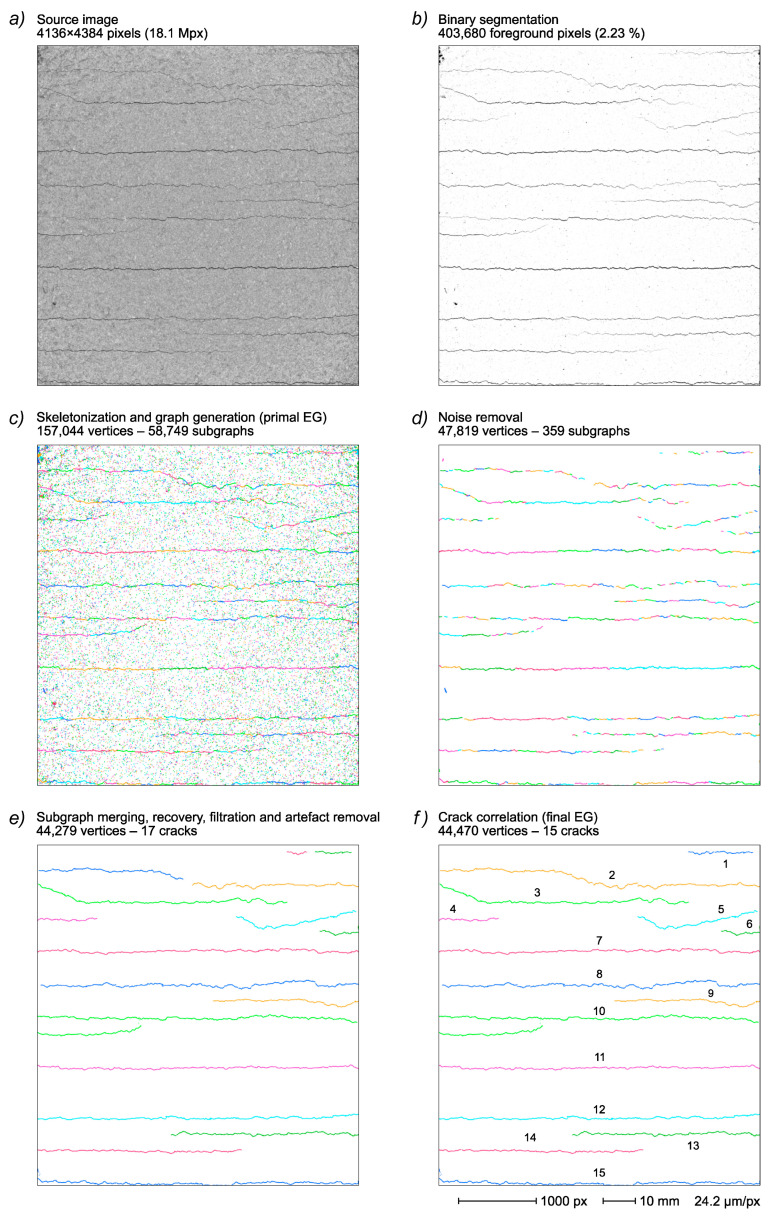
Complete final EG generation from a 1.5% strained sample image. Original image (**a**); binary segmentation (**b**); primal EG after skeletonization (**c**); noise removal (**d**); subgraph merging, recovery, filtration and removal of crack artefacts (**e**); final EG after crack correlation (**f**). Colors indicate different subgraphs/cracks. Distal edges not shown.

All of the reported results were obtained by studying the cracking of reinforced cementitious render samples under a tensile strength test (Section 2). The specific sample image used in the main discussion and the principal steps of the final EG generation are reported in Figure 4.

Even with the quite simple algorithms presented in this work, the adoption of an EG-based crack pattern description can provide interesting enough results that are illustrated and commented on in the following sections. In particular, the advantages are discussed in comparison with the usual implementation based on the direct analysis of the binary-segmented image.

### 3.1. Grayscale Image Segmentation

In the present work, the sole grayscale image preprocessing step is the cutting of original image edges in order to include the sample surface only. It is, however, important to note that image preprocessing methods that can interfere with particular crack measurements (such as crack width) may be safely used here because critical quantifications requiring image access will be carried out later on the original image, using the obtained EG as a guide for accurate crack localization.

A 4136 × 4384-pixel, 8-bit grayscale image of a 10 × 10 cm sample area is cut from the original 6576 × 4384-pixel image of a reinforced render sample strip loaded with 1.5% strain (Figure 4a). The image shows a series of cracks on a rather textured background because of the rough surface of the sample. The resulting binarized image (Figure 4b) has more than 2.2% foreground pixels (404 × 10^3^ pixels versus 18.1 × 10^6^ total pixel count). While demonstrating a reasonable compromise between crack capture and noise rejection, the binarized image retains a significant portion of the noise and, at the same time, exhibits a notable crack fragmentation.

In this work, the segmentation is implemented with threshold binarization, a classical and well-known process with a wide coverage in the literature [42,43]. The determination of appropriate threshold levels is a typically critical task, and several dedicated algorithms were proposed in the literature [44,45,46,47], even specifically tailored for material analysis [34] and crack detection [27,28]. In the case of crack detection algorithms, especially in images with a highly textured background such as in the present study, the threshold level determination is subject to an inevitable tradeoff between the completeness of crack identification (that requires a high sensitivity threshold) and background noise rejection (that requires a low sensitivity threshold instead). However, the use of EGs in crack description allows to add successive EG refining steps for noise rejection and the recovery of lost details.

The whole process thus appears quite robust in respect to the binary thresholding alone, relaxing to some extent the criticality of the latter and allowing to obtain a final, neat crack EG description that is less dependent on the selected threshold level.

**Figure 5:**
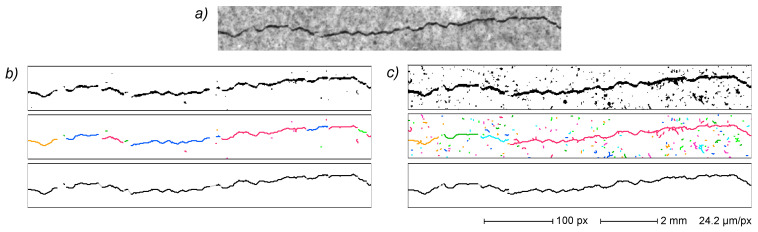
Effect of binarization threshold level. Original image (**a**) and binarization after thresholding at 55% (**b**) and 75% (**c**) of reference intensity level (the central value of the most significant peak in image histogram). For groups (**b**,**c**): binarization (top); primal EG (middle); final EG (bottom). In the final EG images, distal edges are depicted in gray.

An example is given in Figure 5, where the same original image of a cracked surface detail (Figure 5a) is segmented by using two different threshold levels (namely, 55% and 75% of the central value of the most significant image histogram peak, i.e., the most common intensity value of the sample surface). The first set (Figure 5b), acquired at the lower threshold level, shows a small amount of binarization noise in the segmented image (top frame), but also a fragmented crack acquisition that translates to a very fragmented primal EG (middle frame). The second set (Figure 5c), acquired at the high threshold, shows conspicuous noise in both the binarized image and the primal EG but a less fragmented crack in the binarized image, and thus also in the resulting primal EG. However, despite quite different primal EGs, the final EGs resulting from both image segmentations (bottom frame in each set) show a fairly good similarity, with complete noise rejection and nearly identical crack path reconstruction. EG processing can thus compensate, to a rather large extent, the effects of threshold level variations. Moreover, it is worth noting that all crack fragments depicted in the primal EG of Figure 5 are assembled into a single crack subgraph in the corresponding final EG. The threshold level effect is, however, apparent in the end part of very thin cracks, where slight variations could deeply influence the acquisition of cracks’ extreme parts (Section 3.3.7).

Given the above considerations, in the present study a simple global binarization threshold level is thus used for EG generation, and it is computed as 65% of the central value of the most significant peak in the whole image histogram. This results in a fairly complete but noisy and somewhat fragmented crack capture represented by a binary image segmentation (Figure 4b).

### 3.2. Primal EG Generation

The obtained binarized image is then skeletonized (i.e., transformed in one-pixel-wide paths representing the centerline of the binarized objects), which is a key step in order to allow the computation of the crack EG. Skeletonization algorithms are of central importance in image processing and computer vision, with numerous approaches described in the literature [34,48,49,50]. However, there is generally no single ideal skeleton for a given object [51], but a detailed analysis of the skeletonization process optimization for crack analysis was outside the scope of the present study. In this work, the image skeletonization is performed by a thinning algorithm based on a 3 × 3 window applied in four sub-iterations for each cycle [52], resulting in a skeleton of the binarized image composed of 8-connected foreground pixel clusters.

**Figure 6:**
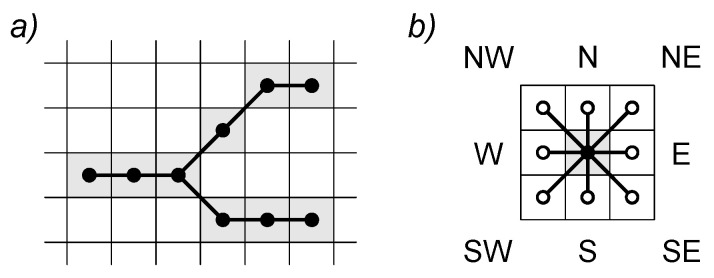
EG generation from skeletonized bitmap. Primal EG vertices (black dots) are uniquely correlated with skeleton pixels (depicted in gray) and are thus constrained to image pixel grids (**a**); edges in primal EG connect only vertices related to adjacent pixels (empty dots), here identified with compass points (**b**).

From the 8-connected skeleton, it is possible to generate a primal EG that completely describes the skeleton path, retaining all the related information. In the primal EG, each vertex corresponds to a skeleton pixel and each edge corresponds to a neighboring pixel connection (Figure 6).

A graph generation process constituted by a scan routine that looks for foreground pixel clusters in the skeletonized image and a subgraph building algorithm (see Supplementary Information S1 for a detailed algorithm description) are used for this study. At the end of the image scanning process, a primal EG is obtained, populated with a complete description of the skeleton pattern as a series of subgraphs, each corresponding to a pixel cluster in the skeletonized image. It is worth noting that the primal EG is characterized by having all vertices corresponding to specific pixels in the original digital image and, consequently, with coordinates specified by two integer values. In the present work, this characteristic is preserved throughout all the primal EG processing; therefore, in the final EG, each vertex also is related to a specific image pixel. This is not by any means a strict requirement and some processing algorithms may result in EGs with vertices specified with non-integer real numbers and therefore no longer traceable to a specific image pixel (e.g., the EG smoothing procedure described in Section 3.4.3). Moreover, all of the edges in the primal EG are established between 8-connected pixels and consequently all the edge lengths are either 1 or √2 (in pixel units). It can thus be useful to distinguish between proximal edges (i.e., edges that connect two neighboring pixels in the primal graph, directly derived from the binarized image skeleton) and distal edges (i.e., edges of length ≥ two pixels that connect two vertices that are not adjacent and that result from subsequent subgraph merging operations).

In the sample in Figure 4, the skeletonization process decreases the foreground pixel count to 157 × 10^3^ (i.e., 39% of foreground pixels present in the original binarized image). The resulting primal EG, directly derived from the skeletonized image, retains all skeleton features yielding a graph with 157 × 10^3^ vertices distributed in 59 × 10^3^ subgraphs (Figure 4c).

The structured data contained in the EG description of the crack path (e.g., the location of end and branch vertices, vertex connection, subgraph identification, and so on) are now readily available for all of the following processes without any further exploration of the skeletonized image. This is a distinctive advantage of the EG representation in comparison to the direct analysis of the binary image, where this information must be retrieved each time by scanning the image itself.

### 3.3. Primal EG Finishing Process

The quality of the crack pattern description given by the primal EG (i.e., completeness, absence of noise or artefacts and so on) is strictly dependent on the upstream segmentation and skeletonization processes. Moreover, specific analyses may have particular quality requirements (e.g., crack count can be heavily degraded by false positives, missed crack detections and discontinuous crack capture). In order to provide an effective functional description of the cracks appearing in the image, the primal EG must therefore be refined with a specific finishing process which includes, among the other things, noise filtering, artefacts elimination and crack merging (when there is evidence that apparently separate cracks are actually parts of a single, extended crack). Primal EG elaboration is thus of central importance, and for the purposes of this work, an automated process is developed based on a series of successive noise elimination and crack correlation steps that take advantage of both the geometrical and the topological properties of crack EG (Figure 3b). In particular, a first noise removal step is followed by a subgraph merging and then by a recovering step in order to reconstruct the full cracks from the sparse subgraph fragments delivered by the binarization process. A second filtration process and an artefact removal step provide for a neat description of the crack pattern. A final correlation analysis searches for cracks that, while separated, are presumably part of a larger crack developed under the sample surface.

#### 3.3.1. Noise Removal

Noise removal is the first step of the primal EG refining process. As previously discussed, the binarization of noisy images inevitably produces a considerable number of false positives (i.e., foreground pixel clusters uncorrelated to actual cracks), which can be challenging to avoid in the first place and to filter out in the post-binarization process. False positive foreground clusters subsequently translate to crack-unrelated subgraphs in the resulting EG that must be identified and removed.

**Figure 7:**
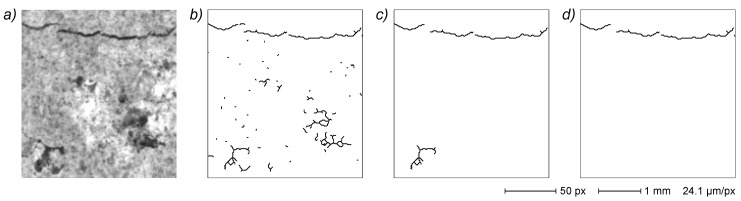
Noise removal with EG processing. Surface texture (**a**) generates a substantial number of noise artefacts in primal EG (**b**) that is mostly removed by the first noise removal step (**c**). The remaining artefact (bottom left) is eliminated in the following sugraph filtration step (**d**).

An example of a noisy sample is given in Figure 7, where the primal EG shows a quite high level of noise-derived artefacts. In particular, dark zones in the original image generate highly branched subgraphs that can easily include more than a hundred vertices. At this stage, this rules out a simple filtration based on vertex count, requiring instead a more specific approach based on geometrical and/or topological properties. Background noise due to sample surface texture is typically characterized by small and compact pixel clusters that originate small-diameter, highly branched subgraphs. A two-step noise removal process is thus implemented in this work. The first step eliminates all subgraphs with a diameter of less than 25 px (defined as the separation of the most distant end vertices). The second step eliminates all subgraphs with a ratio between the total number of vertices and the diameter higher than 2 px^−1^. This remarkably simple process effectively removes the most part of noise artefacts (Figure 7c). A subsequent noise removal step is performed at a later stage of the process through subgraph filtration which further refines the crack EG (Figure 7d).

In the sample depicted in Figure 4, the described noise removal process results in the deletion of >109 × 10^3^ vertices (>69%) and >58 × 10^3^ subgraphs (>99.3%), leading to a near complete noise elimination but substantially retaining almost all crack features (Figure 4d). After noise removal, the EG comprises 47.8 × 10^3^ vertices grouped in 359 subgraphs.

Because of the EG representation, which makes all vertex coordinates and all connections readily available, it is computationally simple to find the end vertices (looking for degree-1 vertices in the EG structure). Therefore, calculating both the subgraph diameter and the diameter/vertex count ratio is a relatively inexpensive process. The same analysis carried out directly on the skeletonized image instead requires, for each foreground pixel cluster, its localization on the image itself, the localization of all the related end pixels by tracking the cluster path and the computation of the total pixel count. This is a relevant consideration due to the high number of subgraphs (tens of thousands) typically present in the primal EG of large images.

A possible alternative to the specific search for noise-related pixels is to process the whole binary segmented image directly with morphological operators (e.g., erosion followed by dilation) in order to eliminate small isolated pixel clusters and then reconstruct the original shape of the remaining image objects [17]. The main disadvantage of this approach is the likely introduction of artefacts into the binarized image (with possible disturbance of subsequent analyses), which practically limits the application of the method to the elimination of very small parasitic clusters.

#### 3.3.2. Subgraph Merging

Typically, the same tradeoff in the binarization process between complete crack description and background noise removal also leads to an incomplete crack capture in the binarized image, resulting in a fragmented crack graph. Separated subgraphs belonging to a single crack must then be identified and joined. This latter task is accomplished by the introduction of new edges between the relevant vertices in the involved subgraphs. There are several ways to identify the subgraphs to be joined. As an example, Ammouche et al. [17] developed an algorithm based on end-to-end crack distance and relative crack angle. In the present implementation, the crack subgraphs are correlated at this stage by simply connecting all different subgraphs with at least two end vertices at a distance ≤ 100 pixels. The actual connection edge is inserted between the two nearest end vertices.

An example of incomplete capture of crack pattern is reported in Figure 4d, where the relatively clean representation of the crack paths demonstrates a deep fragmentation of each crack into several subgraphs (depicted with different colors) because of the small gaps formed during the binarization process. The above-described process provides a quite remarkable result, decreasing the count of subgraph from 359 to 25 and collating most of the visible cracks.

As in the noise removal step, the EG representation eases the process’ computational requirements thanks to the quick localization of end vertices in the graph structure in contrast with the specific pixel search needed when operating on the binary segmented image. Moreover, the EG description allows a straightforward merging of subgraphs by means of edge insertion instead of the morphological manipulations needed for connecting discontinuities in pixel clusters directly on the segmented image [8].

#### 3.3.3. Subgraph Recovering

During the noise removal process, several small subgraphs that belong to genuine cracks are inevitably filtered out. Subsequently, the formerly described subgraph merging step introduces large distal edges in place of the removed subgraphs. The latter can then be recovered by performing a specific search on the primal EG, looking for eligible subgraphs located in the corresponding surroundings of each distal edge. Details of the implemented algorithm are reported in Supplementary Information S2. The subgraph recovery process can substantially improve the quality of the crack EG, retrieving most of the bona fide crack subgraphs filtered out by the first noise removal step.

**Figure 8:**
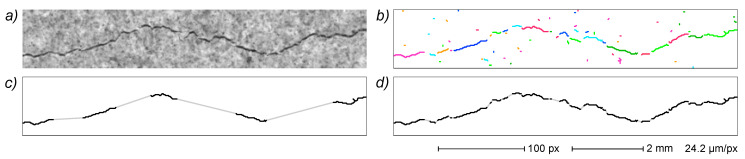
Small subgraph recovery. Image of a small crack (**a**) generates a primal EG (**b**) that is processed up to the subgraph merging step (**c**), where several parts were filtered out during noise removal and replaced by distal edges. The recovering algorithm retrieves the missing small subgraphs from primal EG and inserts them in the merged subgraph (**d**). Distal edges are depicted in gray.

A particularly severe example of subgraph loss is depicted in Figure 8, where a small crack image resulted in a very dispersed collection of small subgraphs (Figure 8b), with large parts canceled afterwards by the noise removal algorithm and thus substituted by distal edges by the subgraph merging process (depicted in gray in Figure 8c). The subgraph recovery process finds the legitimate small crack subgraphs in the primal EG and inserts them into the merged subgraph, with near complete reconstruction of the crack recorded in the original image (Figure 8d). It is, however, important to note that this process is aimed at the recovery of deleted subgraphs in the internal part of a crack path and it cannot retrieve missing small subgraphs located in the crack ends (Section 3.3.7).

In the sample depicted in Figure 4, the subgraph recovery step significantly improved the crack EG completeness, recovering >2300 vertices that add to the >47 × 10^3^ vertices (+5.0%) previously present in the EG.

As in the previous examples, the EG crack description allows an easier implementation of the subgraph recovery algorithm that, in the case of the analysis carried out on the skeletonized image, should instead be implemented with searching processes at the pixel level and image manipulations. A possible alternative is to operate directly on the whole segmented binary image with morphological operations to fill discontinuities in proximal pixel clusters [8]. However, as previously noted, this approach has the disadvantage of likely introducing artefacts into the image itself that may interfere with subsequent analysis.

#### 3.3.4. Subgraph Filtration

After the merging and recovering procedures, the EG is composed of an array of subgraphs, each one related to a significant crack in the original image. Some noise artefacts that are undetected by the former filtering process and some cracks that are too small to be significant can still be present in the EG, however. At this analysis stage, it can thus be useful to implement a further filtration step in order to fully clean up the crack EG. Considering that all of the relevant crack parts are already grouped into large subgraphs, at this stage a simple subgraph filtering step based on the vertex count is applied, eliminating all subgraphs with fewer than 200 vertices.

This yields in the example given in Figure 4 an EG composed of 17 subgraphs (with mean vertex count > 2900), each corresponding to a genuine crack.

#### 3.3.5. Crack Branching and Artefact Removal

Depending on the objective of the whole analysis, it can be useful to identify the main path and the secondary branches of each crack for separate evaluation or for morphological assessment. Moreover, the skeletonization process can generate artefacts due to local variations in crack thickness in the form of parasitic short branches that protrude from the skeleton path [50]. These translate into factitious short arms in the derived crack EG that must be eliminated (pruning). Additionally, spurious cycles are generated during the skeletonization if a small background pixel cluster in the segmented image (or even an isolated pixel) is completely surrounded by foreground pixels. The use of EG-based crack descriptors allows the implementation of the morphological identification of crack path features based on geometrical and topological criteria that can be finely tuned according to the ongoing analysis. Specific features can then be reliably identified on the crack path for further analysis or removal.

In the present case, all sample cracks are produced by the application of unidirectional loading and are therefore characterized by low branching and by the absence of random map, grid-like features [41]. Consequently, the resulting EG pattern description can contain some branches but no significant cycles. Assuming that all cycles present in the EG are therefore due to the skeletonization process and must be eliminated, an artefact removal algorithm is adopted that identifies the main path and the relevant secondary branches, eliminating all residual cycles. The algorithm is based on a series of consecutive weighted shortest path searches based on Dijkstra’s algorithm [37,53] that find the main crack on each sub-EG and then the secondary branches in decreasing length order (see Supplementary Information S3 for complete algorithm description). The iterative procedure is terminated when a minimum branch length is reached (100 pixels) in order to filter out the small parasitic features derived from skeletonization and possible very small, negligible crack branches. The shortest path search also eliminates all subgraph cycles, retaining only the most direct end-to-end path.

**Figure 9:**
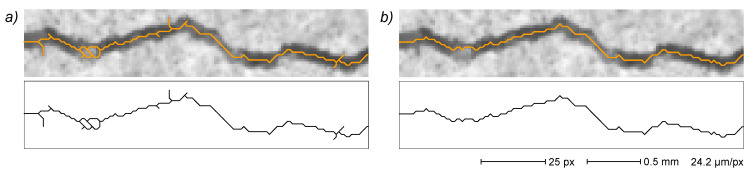
Artefact removal. The EG of a relatively thick crack demonstrates small branches and cycle artefacts derived from the skeletonization process (**a**). A removal algorithm based on shortest path search effectively removes all artefacts leaving the main crack path only (**b**). Top: original image with superimposed EG; bottom: EG alone.

As an example, a typical crack EG containing short parasitic side branches due to noise or local variability in crack width is shown in Figure 9a. The same crack EG also contains small cycles derived from spurious background pixels in the binarized crack image. The previously described procedure allows an effective elimination of all artefacts (Figure 9b), resulting in a neat, unbranched crack path.

In the complete image analysis reported in Figure 4, the same process removed ~5300 vertices (10.7%), effectively cleaning the crack EG (Figure 4e).

The advantages of implementing the crack path description by EGs become apparent when considering alternative approaches based on the direct analysis of the binary-segmented image. A thorough description of the morphological separation of branching cracks in binary images is given by Arena et al. [35]. In this example, the separation is obtained with a quite elaborate procedure that includes several morphological operations for the localization of the crack connections (carried out on a separated copy of the image) and then the final separation of each crack path in the original image by erasing parts of the crack pattern at the branching point. Another approach to the problem posed by the separation of connected crack paths is to implement a series of morphological operations directly on the segmented image (e.g., erosions and dilations) in order to detach each crack into an independent cluster and then reconstruct in the best possible way the original image [18]. The major disadvantage of these approaches is the high computational cost and, above all, the manipulation of the segmented image with the possible introduction of artefacts and consequent errors in the following analyses. Considering the pruning process instead, several algorithms working directly on skeleton images are described in the literature [50], but the EG representation also enables the simultaneous processing of parasitic cycles and the identification and classification of secondary branches, as previously described. Moreover, the described approach based on EGs avoids the apparent crack length reduction derived from some pruning methods performed directly on the skeleton image, such as cutting pixels from each crack branch endpoint [33]. On top of that, it is worth noting that the graph representation allows a great optimization of the computational cost related to the path search algorithm by substituting the complete crack EG with an equivalent, smaller representation based on weighted graphs from which all degree-2 vertices are removed (Supplementary Information, Section S3.2). This is particularly effective for crack EGs, because they consist mostly of quite long chains of degree-2 vertices (e.g., the crack EG of the sample reported in Figure 2 comprises, at this stage of the analysis, 49,577 vertices subdivided in 1682 degree-1 vertices, 45,999 degree-2 vertices and 1896 degree-3 vertices).

#### 3.3.6. Large Crack Correlation

Depending on the specific purpose of the analysis, each identified crack can be considered as independent from the others, or it may be advisable to perform a search for possible correlations of contiguous cracks. As an example, in the laboratory samples measured in the present work, large cracks are typically generated perpendicularly to the strain vector at the reinforcing fibers. Consequently, it is of great convenience to consider all contiguous cracks formed along these preferential directions as parts of bigger cracks. The availability of an EG-based crack description allows the implementation of several algorithms for the evaluation of possible correlations between different cracks included in a single image. This study uses a correlation search algorithm based on the relative orientation (<45° angle) and distance (<10 mm) of subgraph end parts (see Supplementary Information S4 for details), analogous to the criteria adopted by Ammouche et al. [17] for crack correlation in microscopic images.

In the reported sample, the processing of the crack EG with the described algorithm allows to identify two cases (corresponding to the four upper cracks in Figure 4e) and, after a subgraph joining operation, the resulting final EG is composed of 15 subgraphs, each correlated with a single crack. Moreover, during the joining process, a further search for the recovery of small subgraphs (as described in Section 3.3.3 for distal edges) is carried out, resulting in 191 added vertices.

While a very similar algorithm can be operated directly on a binary image [17], the EG crack description enables an easy implementation by making vertex positions and connectivity data readily available.

#### 3.3.7. Final EG Evaluation

The resulting final EG (Figure 4f) is constituted by >44.4 × 10^3^ vertices distributed in 15 subgraphs and represents a quite good description of the crack pattern recorded in the original image.

**Figure 10:**
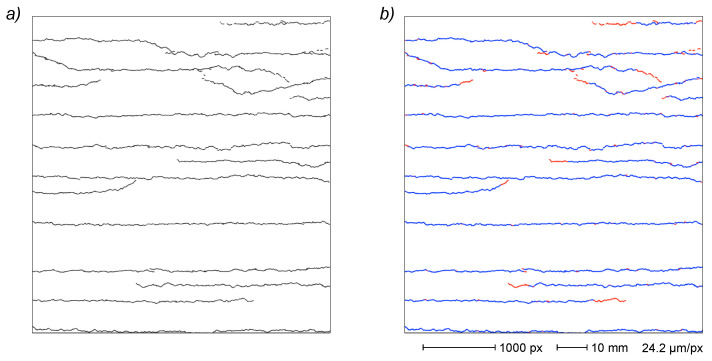
Reference ground truth EG for the sample depicted in Figure 4 manually derived from the original sample image (**a**). Ground truth with identified (blue) and not identified (red) crack paths in the automatic crack processing results depicted in Figure 4f (**b**).

The quality of final EG as a crack path descriptor is evaluated using an EG-based reference ground truth (Figure 10) manually obtained from the same image depicted in Figure 4a (Section 2.2). Two different EGs can be quantitatively compared by determining, for each of them, the part of the path that overlaps with the other. EG overlap is defined as the part of the EG path that has no distance from the path of the other EG greater than a given tolerance. The EG–EG comparison thus allows to identify the overlapping part of the final EG and the ground truth EG (true positives, TP), the non-overlapping part of the final EG (false positives, FP) and the non-overlapping parts of the reference EG (false negatives, FN). The TP, FP and FN are quantified as path length on their respective EG. The values obtained are then used for the EG-based computation of well-known performance evaluation indicators, namely precision, recall, intersection over union (IoU) and F1-score quality measures. The detailed EG path comparison and performance evaluation methods are reported in Supplementary Information S5. The same evaluation process is carried out also for primal EG. Both EG–EG comparisons are carried out with 100 μm (4.13 px) overlapping tolerance (results are reported in Table 1).

The labeled ground truth with identification of the crack parts recognized by the automated process (i.e., overlapping with the final EG) is shown in Figure 10b. The comparison indicates that no cracks are missed, with the main path of all of the cracks reported in the original image correctly identified in the final EG. The comparison of the primal EG and final EG quality measures quantifies, at the crack path level, the results of the primal EG finishing process shown in Figure 4. The high noise level in the primal EG (Figure 4c) is reflected in the very poor precision and IoU values (51.5% and 51.3%, respectively) and in the low F1-score (67.8%). However, the recall value is quite high (99.2%) thanks to the threshold setpoint aimed at capturing most of the crack details. The huge improvement from this starting point to the final EG (Figure 4f) is testified by the substantial gains in the precision and IoU demonstrated by the latter (increased to 99.5% and 91.7%, respectively). However, the same process causes a small but significant loss in the recall value, which instead decreases to 92.2%. The primal EG processing action may thus be seen as a fair compromise between noise removal and the completeness of crack path capture, where a large gain in precision and IoU is obtained at the cost of a small decrease in the recall value. This is effectively summarized by the important F1-score value increasing to 95.7%.

In greater detail, the lower recall value in the final EG is due to missing crack parts related to very thin features mostly located on crack ends (Figure 10b). This is primarily caused by the heavy fragmented crack pattern detection in low-contrast, very low-thickness crack zones that are filtered out in the noise removal step and are not retrievable by the simple recovery algorithm implemented in this work (Section 3.3.3). Despite the described issue, the reported polishing process, even if implemented with relatively simple algorithms, demonstrates the advantages of EGs for the generation of a neat crack pattern description, notwithstanding the intrinsic limitations of the crack detection based on the global threshold binarization of a noisy image.

The refined EG is then available as a basis for several different EG-based crack analyses, some of which are reported in the following sections.

### 3.4. Crack Analysis

The obtained final EG gives an effective functional description of the crack pattern on the sample surface, with each crack individually represented by a connected sub-EG. As previously noted, with the implemented procedure, all the final EG vertices retain the strict correlation with the image pixel from which they are derived, and they are thus localized with single pixel precision. By describing the crack paths with a high resolution, the final EG allows direct EG analyses (e.g., for crack length determination), EG-driven analyses on the original image (e.g., for crack width measurement) and crack correlation analyses in time-series images (e.g., for crack evolution studies).

#### 3.4.1. Selection of Cracks and Measurement Zones

In the analysis of cracked surfaces, it can be convenient to select particular cracks and even specific crack parts to be measured according to the objectives of the analysis itself. As an example, during crack width measurement, it is usually appropriate to avoid intersection zones. It is possible to eliminate these disturbing features directly on the binarized image by using a series of cleverly arranged morphological operations on the image itself, as described by Soroushian et al. [18]. Nevertheless, the availability of EG description allows an effective analysis of the crack pattern by geometrical and topological algorithms, enabling a rational selection of the cracks or the crack parts to be measured using objective criteria and avoiding the introduction of possible artefacts due to manipulations of the binarized image. In the aforementioned crack width measurement, the identification of the potentially interfering zones such as crack ends or intersections can be easily accomplished on the crack EG by selecting the internal vertices that are beyond a minimum stated distance from end vertices or from branching vertices (Section 3.4.7).

#### 3.4.2. Direct EG Measurements

In the present work, the position of each EG vertex is determined from the skeletonized image and never modified (the implemented primal EG processing methods can delete vertices but not change their coordinates). Therefore, the actual positional precision of the crack pattern in the final EG depends directly on the implemented image segmentation and skeletonization procedures. Several algorithms were proposed in the literature both for binarization [27,28,34] and for skeletonization [48,49,50,51], but a detailed study of their effects on crack tracking errors is outside the scope of the present work. However, in considering that most skeletonization issues are related to the thinning of thick image features [4] and that the typical crack thickness analyzed in this work is a few pixels, the skeleton obtained from the image segmentation (and thus the EG derived after the removal of skeletonization artefacts) is regarded as a reliable description of the crack pattern. The final EG can therefore constitute the basis for the direct measurement of several path-derived crack properties. Some of these can also benefit from an EG smoothing procedure in order to alleviate the errors due to the spatially discrete nature of the original digital image representation.

#### 3.4.3. EG Smoothing

As mentioned above, all vertices in the final EG are bound to specific pixels in the original digital image and are therefore localized by discrete spatial coordinates. Consequently, even if the EG path of a horizontal, vertical or 45° diagonal line is constituted by a series of aligned vertices, paths at every other inclination generate rough EGs composed of a saw-edged succession of short segments. The EG of a generic curve is thus represented by a discrete approximation made from a jagging path through the image pixel grid. This can lead to errors in some measurements carried out on pristine EG (e.g., in path length determination, where each EG deviation from the actual path causes a small positive error that accumulates in the process). This problem can be addressed by using a smoothed EG in order to perform critical measurements with a more even path.

In this work, a simple but effective iterative algorithm for EG smoothing (described in Supplementary Information S6) is implemented that acts on internal vertices, leaving all end vertices and all branching vertices untouched. It is worth noting that the described procedure results in a new EG with several vertices that are no longer uniquely related to the original image pixels (i.e., several internal vertices will have non-integer coordinate values).

**Figure 11:**
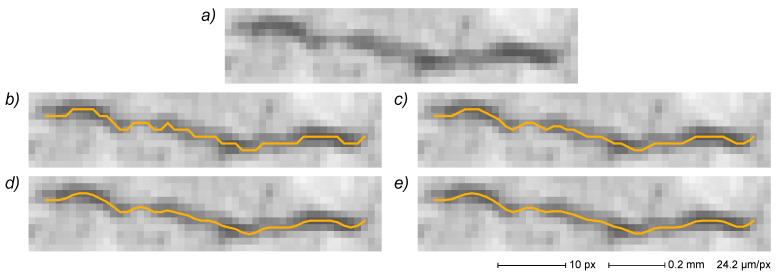
EG smoothing. A crack EG (**b**) is obtained from the original image (**a**) with vertices corresponding to pixel centers, resulting in a quite rough path. Paths deriving from one (**c**), two (**d**) and three (**e**) EG smoothing iterations, respectively, demonstrate a progressive improvement in evenness.

An EG smoothing example is reported in Figure 11 for a small crack image. The original EG (Figure 11b) is characterized by a strong jagging because of the direct correspondence of each vertex with a pixel in the skeletonized image. EGs derived from one, two and three smoothing cycles (Figure 11c–e, respectively) show a progressive improvement in the path evenness.

By comparison, an effective implementation of the described smoothing algorithm directly on the binary-segmented image appears quite difficult at the very least, requiring the creation of an EG-like data structure for the manipulation of pixel coordinates and pixel-to-pixel connections. Moreover, even without resorting to a smoothing process, the direct measurement of crack length on the skeletonized image requires the implementation of a specific algorithm for the localization of each crack starting pixel and for the tracking of the crack path on the image itself [20] instead of the very simple edge length addition performed on the crack EG.

#### 3.4.4. Crack Path Length

For unbranched cracks, the crack length can be defined as the length of the curve describing its centerline. For cracks containing branches or loops, there are several possible length definitions, such as the sum of the lengths of all crack components (i.e., the total path) or the length of the shortest path between the most distant end points (i.e., the length of the main crack path). Depending on the application, it may also be appropriate to include in the length determination all major branches excluding small cycles and minor features or even to evaluate the cumulative length of all crack parts exhibiting a width value greater than a given minimum. As previously discussed, the availability of EG crack description allows to fine-tune the crack parts to be measured according to well-defined selection rules.

In the present work, the crack length is defined as the cumulative length of the whole crack path reported in the final EG (i.e., without artefacts) measured as the sum of Euclidean distances between connected vertices (i.e., the sum of all edge lengths). Moreover, in order to minimize the errors due to the discrete nature of the image grid (from which the EG is derived), in the present study a three-cycle EG smoothing procedure (Section 3.4.3) is used before each length measurement. It is, however, important to note that very thin crack ends can be lost in the EG finishing process (Section 3.3.7), introducing a potential error source in crack length measurements.

#### 3.4.5. Crack Branching

**Figure 12:**
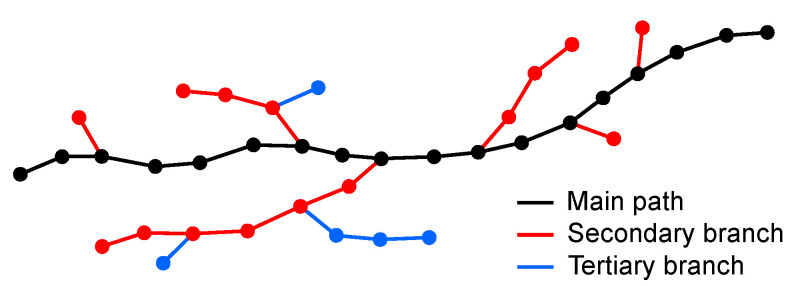
Fictitious crack EG with identified main crack path (black) and lateral branches (red and blue). The latter can also be classified by length or by the connection hierarchy as depicted in the figure.

The analysis of crack branching can be directly derived from the final EG. As described in Section 3.3.5, the same process used for the removal of skeletonization artefacts can also hierarchically order all crack branches, starting from the main path and proceeding through lateral arms in decreasing length order. Furthermore, the described algorithm allows to classify the found lateral branches as primary (if directly connected to the main path), secondary (if connected to a primary branch) and so on (Figure 12). A basic crack branching analysis is thus made available as a by-product of the final EG generation process. Further analyses can then be developed based on specific needs exploiting the EG geometric and topological information.

#### 3.4.6. EG-Driven Measurements on the Original Image

Besides direct measurements, the EG-based description of a crack pattern, expressed in pixel coordinates, also allows a guided crack analysis on the original image. One of the most important applications is the measurement of crack width, that can greatly benefit from the EG description. In fact, such description allows to easily select relevant crack portions to be measured and to implement tailored algorithms for EG-driven width measurement on the original grayscale image (avoiding in this way all possible artefacts generated in the subsequent image processing for crack detection).

#### 3.4.7. Crack Width Measurement

**Figure 13:**
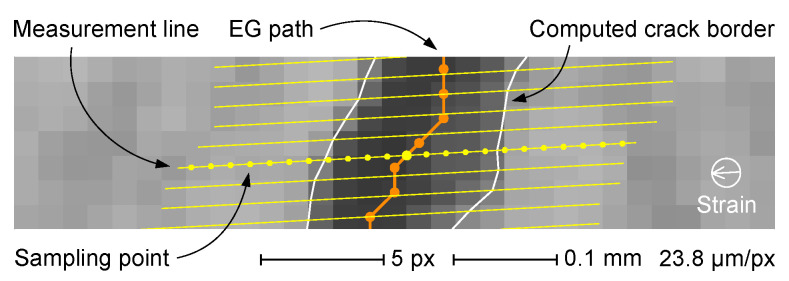
EG-driven crack width determination on the original grayscale image with parallel measurement lines along the strain direction. Measurement lines (yellow) shown here are very short for the sake of clarity (typical sampling point count > 200). The center of each measurement line (thicker yellow dot) is located at the intersection with the crack EG path (orange). The distance between sampling points (yellow dots) and between measurement lines is 0.8 pixel.

The crack width can be defined globally for the whole crack or locally at a given crack path point, with various image-based measurement procedures described in the literature. As an example, in the former case, the mean crack width may be evaluated by dividing the entire crack area by the measured crack length [33,35]. In a comparable way, the local crack width can be derived from binary segmented images isolating the involved neighboring crack area by intersection with a strip kernel normal to the local crack tangent and then dividing the obtained area by the kernel width [36]. The local crack width can, however, be defined in several other ways, such as the crack boundary-to-boundary minimum distance [23] or twice the minimum crack boundary distance from a given point of the central line [7]. The definition of a local crack width, giving the best results in most contexts [4,7], is the distance of the intersection points of the crack boundaries from a line perpendicular to the tangent of a given point of the central line [54]. With crack EG availability, this method can be implemented very conveniently by computing the tangent line of a given EG vertex by linear fitting of neighboring vertex positions. The crack width is then evaluated on the original image along a measurement line perpendicular to the tangent line and passing through the involved vertex. A slightly different approach that is appropriate, for example in the case of constant strain direction, entails the use of parallel measurement lines at a fixed orientation. Crack EGs also make this approach very practical, as it may be implemented by defining a series of parallel equidistant lines and then finding the intersection point between these latter lines and the EG itself (Figure 13).

All crack examples analyzed in this work are recorded in samples under tensile test (i.e., subjected to uniform strain condition). Consequently, the parallel lines approach is adopted, using fixed-length measurement lines oriented as the strain vector. The crack width determination along an arbitrary measurement line requires the extraction of the original image intensity values in a direction typically unrelated to an image axis (i.e., not oriented along the digital image grid). This can be achieved by locating a series of sampling points at constant intervals on the measurement line itself (Figure 13) and then computing the interpolated intensity value for each point from the neighboring image pixels. The bilinear interpolation of the intensities of the four pixels nearest to the sampling point is used in this work. The described procedure generates a series of sampled values (an intensity profile) in which the central value belongs to the crack because it is located on the EG crack path (being at intersection of this latter and the sampling line itself). Measurement of the local crack width subsequently translates to the determination of the crack edge position on the intensity profile obtained from the original image. This can be a challenging task in noisy images, and, for the purpose of this work, a specific algorithm is implemented based on the determination of a local threshold for each intensity profile (see Supplementary Information S7 for a detailed description of the crack edge localization algorithm).

**Figure 14:**
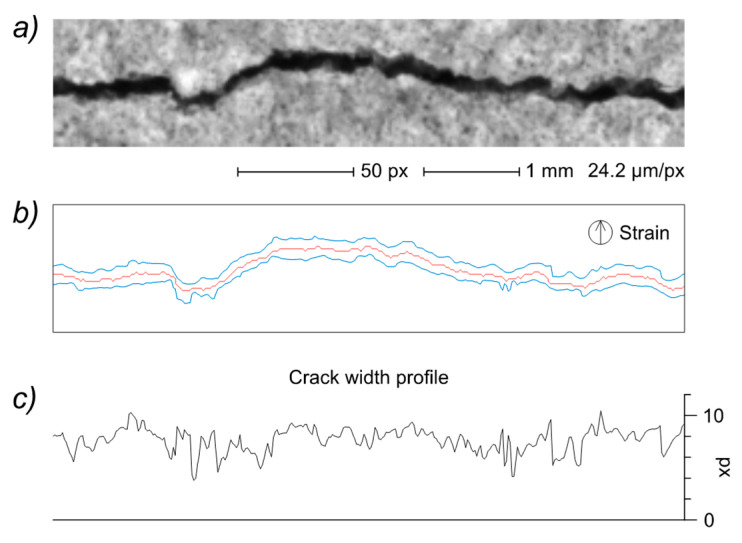
Crack width profile (detail) computed on a crack image by EG guidance. Original crack image (**a**); crack EG (red), computed crack edge (blue) and strain direction (**b**); crack width profile computed along the strain direction with parallel measuring lines (**c**).

In this study, the crack width measurements are carried out with parallel measurement lines oriented along the strain direction and separated by a constant 0.8-pixel distance. Each local width measurement is performed starting from the intersection of the respective measurement line with the crack EG (Figure 13) and taking a series of sampling points on the original grayscale image at a fixed 0.8-pixel distance. The slight oversampling allows to take full advantage of the image information content. The width of each crack is calculated as the mean of the local width measurements, acquired in selected path zones in order to avoid interferences from disturbing the features (valid measuring points are at least 5 pixels away from end vertices, 10 pixels away from branching vertices and 3 pixels away from distal edges). The availability of the local width measurement also allows to draw the crack width profile as in Figure 14, which demonstrates the high local variability typical of cracks in cementitious materials [7].

While several examples of crack width determination carried out directly on the segmented binary image have been described in the literature [7,22,36], the implementation of the EG-driven measurement on the original grayscale image allows to avoid potential pitfalls due to previous image manipulation processes [27]. More importantly, the availability of an EG-based crack pattern description allows the implementation of tailored algorithms for automatic identification of specific crack zones to be measured or skipped.

#### 3.4.8. Crack Evolution in Time-Series Images

A usual requirement for field and laboratory damage evaluation is the assessment of the temporal evolution of the crack pattern. In these studies, crack images recorded at different times are typically compared in order to assess the progression of crack count and the evolution of length, width and branching of each detected crack.

As an example, the typical render tensile test used in this study (Section 2) produces a series of five images that record the progressive crack development in the specimen with the increase in the applied strain. Cracks appeared and evolved during strain application, but never disappeared because the load is monotonically increased. Moreover, most cracks in these samples develop along reinforcement fibers perpendicular to the strain direction, and crack coalescence is typically observed in the image series.

It is thus very convenient to describe the crack pattern evolution with a genealogy tree, where each crack in different images (i.e., at different times) is represented with a vertex and the crack relationships are represented by edges. This representation allows to model the evolution of the crack pattern, enabling the automated analysis of time-related crack properties. In order to build the crack genealogy tree, it is necessary to identify all crack correspondences in the whole image series. The actual crack association through different images can be carried out on the EG crack representations looking for the best correspondence of the crack patterns (i.e., by correlating the spatial position of the respective EG vertices). Moreover, in the present work, the camera is secured to the test machine so the only recorded crack movements are due to sample deformation during the test. The latter causes the sample surface (and thus the cracks) to move in the strain direction and, considering that the sample bottom side is fixed and the strain is applied to the upper side, all apparent movements are always directed upwards. The search for the correlated cracks in image series is thus performed, seeking, for each crack, the nearest sub-EG in the following image, taking into account the expected movement of the sample surface. This results in an effective crack matching process that allows to reliably reconstruct the crack genealogy tree and to correlate the measured crack parameters to the test progression.

**Figure 15:**
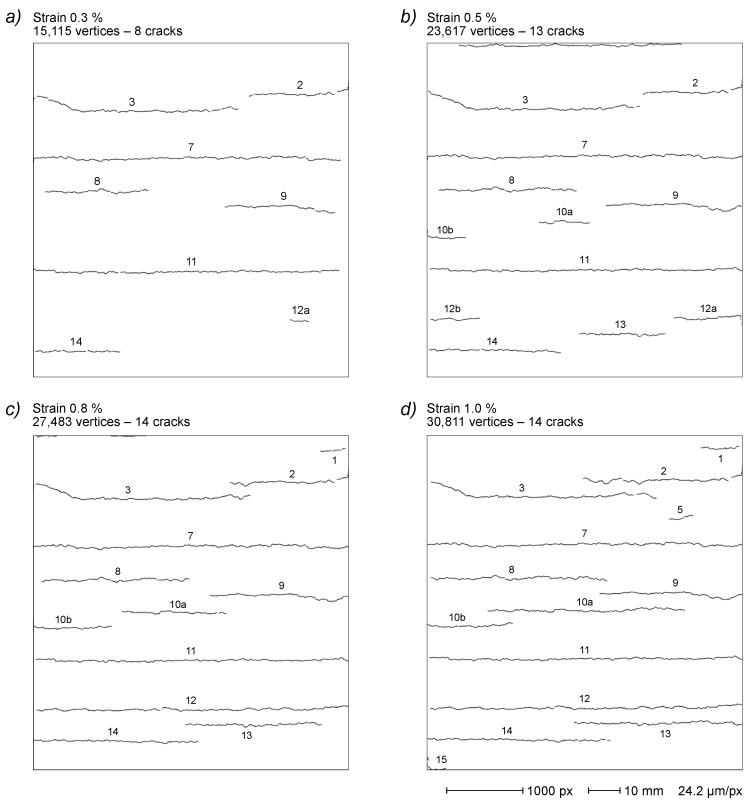
Crack patterns (final EGs) of the sample depicted in Figure 4a at different strain levels (from 0.3% to 1.0%). Cracks labelled as the corresponding cracks at 1.5% strain (Figure 4f).

The image reported in Figure 4a illustrates the final step of the described render strip tensile test showing 15 cracks recorded at 1.5% strain. The complete series comprises four other images acquired at 0.3%, 0.5%, 0.8% and 1.0% strain values, respectively, demonstrating a progressive crack network development with a cumulative increase in crack count, extension and width, also including some cases of crack coalescence (Figure 15).

**Figure 16:**
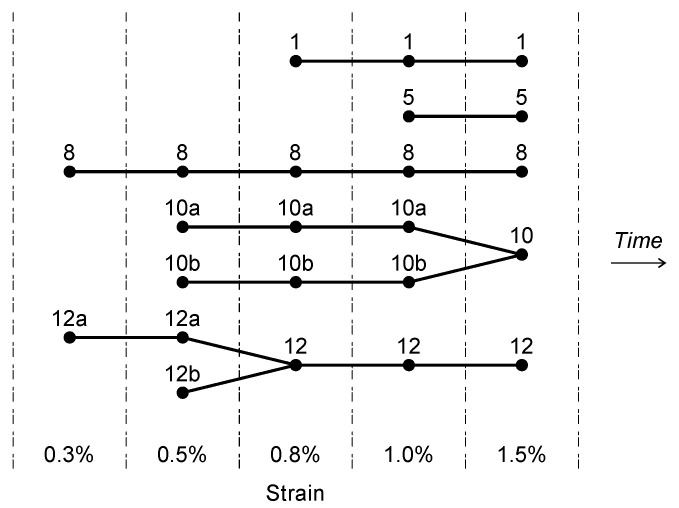
Computed crack genealogy trees for selected cracks in the sample shown in Figure 4 and Figure 15. Each vertex represents a specific crack (sub-graph) in the final EG of the corresponding image. Two crack coalescence events are observed at 0.8% and 1.5% strain. Crack labels as depicted in related figures.

The analysis of the crack evolution results in a specific genealogy tree for each crack recorded in the final step image. The computed trees (Figure 16) show two crack coalescence events at 0.8% and 1.5% strain (for crack 12 and 10, respectively).

**Figure 17:**
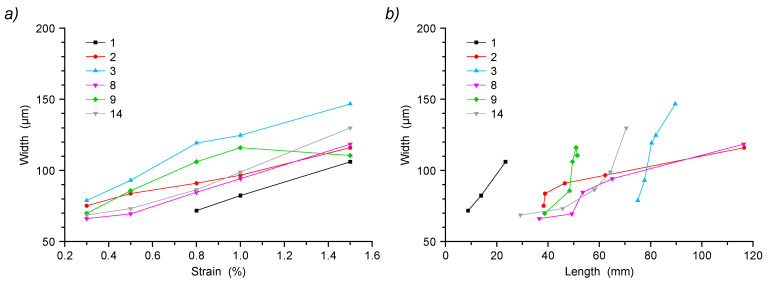
Crack width vs. sample strain (**a**) and crack width vs. length (**b**) for selected cracks in the sample shown in Figure 4 and Figure 15. Width and length values are derived from the pixel values using the image reproduction scale. Points in each set of (**b**) correspond to different steps in strain progression (from 0.3% to 1.5%). Crack labels as depicted in Figure 4f.

The obtained representation enables the automated analysis of time-related crack properties. As an example, the progression of crack width with applied strain is reported in Figure 17a for selected cracks (identification numbers follow the ones reported in Figure 4f). As expected, all cracks demonstrate a progressive width increase with the increase in strain. The only exception is for crack 9, where the proximity to the larger cracks 8 and 10 possibly allows some local relaxation. Instead, Figure 17b shows the evolution histories of the same cracks as the relationship of crack width vs. crack path length. The obtained data show very different evolutions, with some cracks that grow very quickly in length, reaching the full sample width, and others that steadily increase in both width and length.

The previously described enhancements in crack pattern refinement and analysis made possible by the EG-based description allow to increase the reliability of such analyses with interesting potential in the development of systems for automated structural health monitoring. Moreover, the availability of crack EGs allows to perform an effective image-to-image crack correlation using polished path data, avoiding possible issues related to noise and image segmentation artefacts.

## 4. Conclusions

This work proposes a new strategy for the automated analysis of crack images based on the use of EGs as crack pattern descriptors for all post-detection processes. EGs integrate both geometrical and topological information of the detected crack path in a compact representation, facilitating the development of effective algorithms for crack refining, selection, correlation and analyses. The repeated explorations of the bitmap image required with the traditional approach are then replaced by the single initial scan required to generate the primal EG, which then becomes the unique, highly accessible reference for all of the crack path information contained in the skeletonized image. After a finishing process, the required crack analyses can be carried out both directly on the resulting final EG and/or on the original grayscale image using the final EG as a guide (thus avoiding issues related to image manipulations during the identification process).

The described approach offers several other advantages, including the easy manipulation and refinement of the crack pattern, the precise localization of the crack on the original image and the availability of a very accurate functional description of the crack path that is an invaluable resource for documentation and postponed analyses. The obtained EGs can also be evaluated with standard performance metrics, specifically adapted for use with EG crack representations.

Moreover, the EG description allows the automated selection of particular cracks and crack features using objective criteria based on the geometrical and topological features of the EG path, enabling the autonomous execution of specific analyses on selected crack parts (e.g., crack width measurements avoiding crack ends and bifurcations).

Since EGs can be derived from any binary-segmented image, the described strategy can be applied to most crack detection approaches, allowing the implementation of sophisticated post-detection refinements and analyses that would be difficult or impossible to implement directly on the pristine segmented image. The EG representation of the crack pattern therefore constitutes an ideal interface between any crack detection algorithms resulting in a binary segmentation (e.g., threshold binarization and CNNs) and the following post-detection analysis processes.

The effectiveness of the described approach is demonstrated by a challenging case based on the analysis of a laboratory render strip tensile strength test, characterized by a high noise level due to a very pronounced surface texture due to the sample roughness. Moreover, the simple crack detection algorithm adopted, based on the global threshold binary segmentation, provides a raw starting point that requires a substantial amount of EG refinement, illustrating the great potential of EG implementation for noise reduction, crack path reconstruction, artefact removal and crack classification and association. The implementation of an EG-based automated measurement of the evolution of crack length and width is also demonstrated by the unattended identification and avoidance of potentially disturbing features such as branching and end points.

The illustrated procedures are, however, only examples of the possibilities offered by the compact description of geometrical and topological properties conveyed by EGs, and many other opportunities may arise from the combination of existing approaches for image processing with the vast collection of graph-related algorithms and from the development of specialized, EG-guided analyses on the original image. The implementation of EG-mediated analysis processes can thus allow to fully exploit the potential of most of the existing detection algorithms for cracks and other curvilinear features.

## Figures and Tables

**Table 1:** Quantitative quality measures for primal EG and final EG obtained by EG–EG path comparison with the ground truth EG (100 μm overlap tolerance). Definitions and computation details are reported in Supplementary Information S5.

Measure	Primal EG	Final EG
TP ^1^	58,417 px	52,266 px
FP	55,059 px	289 px
FN	489 px	4453 px
Precision	51.5%	99.5%
Recall	99.2%	92.2%
IoU	51.3%	91.7%
F1-score	67.8%	95.7%

^1^ Mean of sample and reference matching paths.

## Data Availability

Data are contained within the article or Supplementary Materials.

## Supplementary Materials

The following supporting information can be downloaded at: https://www.mdpi.com/article/10.3390/s22165942/s1, S1: Euclidean graph generation algorithm; S2: Subgraph recovering algorithm; S3: Artefact removal algorithm; S4: Crack correlation search algorithm; S5: EG–EG quantitative comparison algorithm; S6: EG smoothing algorithm; S7: Crack edge identification algorithm.
