# Supplementary material for: Euclidean Graphs as Crack Pattern Descriptors for Automated Crack Analysis in Digital Images
Source: Sensors (Basel). 2022 Aug 9;22(16):5942. doi: 10.3390/s22165942 (PMC9414651; doi:10.3390/s22165942)
Supplement: Supplementary file 1 [file sensors-22-05942-s001.zip › sensors-1828473-supplementary.pdf]

© 2022 by the Authors.
This document is distributed under the CC-BY 4.0 license.
https://creativecommons.org/licenses/by/4.0/

SUPPLEMENTARY INFORMATION

Alberto Strini and Luca Schiavi
Euclidean graphs as crack pattern descriptors for automated crack analysis in digital images
Sensors **2022**, *22*, 5942
https://doi.org/10.3390/s22165942

S1. Euclidean graph generation algorithm

A Euclidean graph (EG) describing the raw path of cracks or other line-shaped features can be generated from an 8-connected skeletonized bitmap image with complete information retention (*i.e.* with a two-way correlation between the EG vertices and the bitmap foreground pixels). The edges of the generated EG (primal EG) reflect the 8-connected neighborhood of each skeletonized bitmap pixel. The EG generation process is based on the assembly of the sub-EGs built from each connected foreground pixel cluster in the skeletonized image. The process is thus composed of an image scan and a graph building algorithm, the former seeking for unvisited pixel clusters and the latter exploring each located cluster with an exhaustive process analogous to the depth-first search (Figure S1). More in detail, when the scan process locates a foreground unvisited pixel, the graph building procedure begins using the former as initial working pixel. The working pixel is marked as visited, then a corresponding new EG vertex is generated and the 8-connected pixel neighborhood is explored. If an unvisited foreground pixel is located in the working pixel neighborhood it is marked as visited, a new vertex is added to the EG and a new edge is also added, connecting the new vertex with the working pixel vertex. The located pixel will be the working pixel for the next iteration. If more than one unvisited foreground pixel is found, the remaining ones are also marked as visited, their corresponding vertices and edges are generated and added to the EG and the vertices are saved in a temporary buffer for later exploration. If an already visited pixel is found, the corresponding vertex is searched on the EG and, if not already present, a new edge is inserted between the latter and the working pixel vertex. The building procedure terminates when unvisited pixels are no more available (*i.e.* when unvisited pixels are no more present in the current 8-connected neighborhood and no vertices are available in the temporary buffer). The bitmap scan procedure can then be resumed looking for the next unprocessed cluster. The resulting primal EG retains all information from the original skeletonized bitmap, each vertex acquiring the geometrical coordinates of the corresponding skeleton pixel and each edge reflecting its 8-connected pixel neighborhood. It is worth noting that, originating from a digital image, all vertex coordinates in the

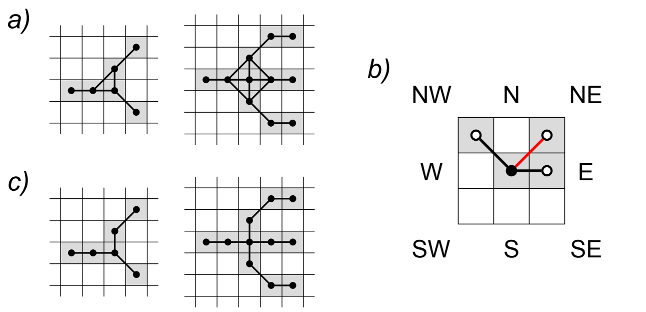

Figure S2. Parasitic 3-cycles (triangles) generated at the skeleton branching points (a). The improved EG generation algorithm (b) adds a diagonal edge (NW in figure) only if none of the two proximal pixels (N, W) is a foreground pixel and rejects a diagonal edge (NE) if one proximal pixel is foreground (E). Resulting EG without parasitic triangles (c).

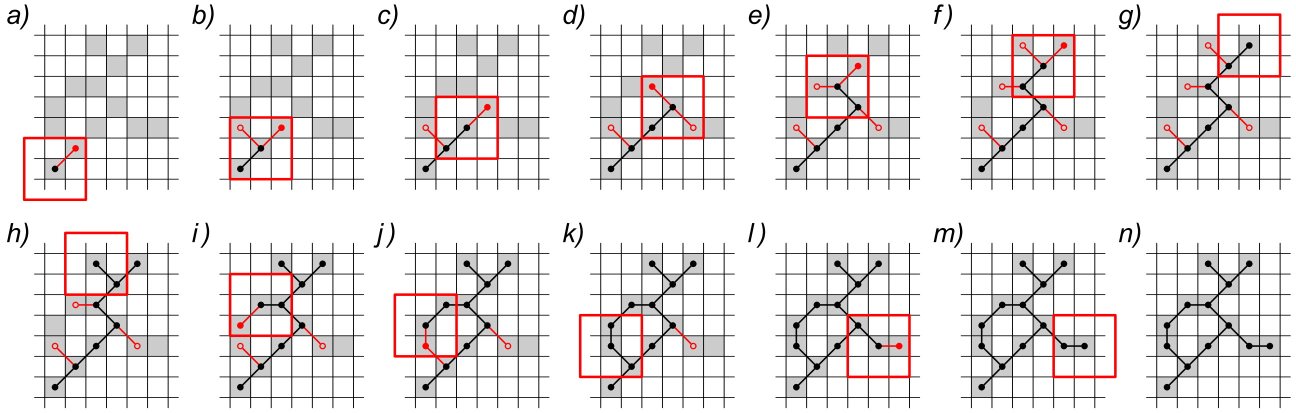

Figure S1. EG generation algorithm from a skeletonized bitmap. A graph vertex (black dot) is generated for a corresponding skeleton foreground pixel (gray squares) and its 8-connected pixel surroundings (red square perimeter) are checked for the presence of other foreground pixels. A new graph vertex (red dot) is generated for each foreground pixel found and a corresponding graph edge (red line) is inserted. The first vertex found will become the next working vertex (red filled dot) while the others are saved for successive exploration (red empty dots). The procedure is iterated until exhaustion of vertices to be explored (a–n).

primal EG inherit the corresponding image pixel coordinates and are therefore represented by integer values.

A possible issue related to the described algorithm is the creation of small 3-cycle artefacts (triangles) in case of some branching pixel configurations, because the building process generates an edge for the 8-connected neighborhood of each pixel (Figure S2a). If this is an undesired behavior (as in the present work), it can be corrected by ensuring that edges in diagonal directions (*i.e.* NE, NW, SW and SE) are added only if the related proximal pixels in the axis directions (*i.e.* E, N, W and S) are not foreground pixels (Figure S2b). This results in an EG without parasitic triangles (Figure S2c).

S2. Subgraph recovery algorithm

Several small subgraphs in primal EG that belong to genuine cracks are inevitably filtered out during the first noise removal process. In the subsequent merging step, large distal edges are then introduced in the working EG in place of the removed subgraph. The latter can be recovered by performing a specific search on the primal EG for each distal edge (*i.e.* with length ≥2 pixels) present in the working EG. Distal edges can result only from the subgraph merging process because all EG edges originating from the skeletonized bitmap connect two neighboring pixels and thus their length is always <2 pixels. The recovery process is implemented for each distal edge as described below. An acceptance circle is established using the distal edge midpoint as centre and the edge length as diameter (Figure S3), after multiplication of the latter by a given constant (1.5 in this work). The enlarged acceptance circle defines the area in which eligible subgraphs are searched in the primal EG. A subgraph is eligible for insertion in the merged graph if it has at least two end vertices inside the acceptance circle. A cost parameter defined as the sum of the distances from the long edge vertices to their respective nearest subgraph end vertices is calculated for each eligible subgraph (provided that each distance is smaller than the distal edge itself). The subgraph with the smallest cost parameter it is selected among the eligible subgraphs (if any). The selected subgraph from the primal EG is then inserted in the merged subgraph by substituting the distal edge with two new edges. In order to avoid introduction of undesired artefacts, only the shortest path between the selected subgraph end points is actually added to the merged subgraph. If needed, this procedure can be iterated by searching for other small graphs to be inserted within the two newly added long edges.

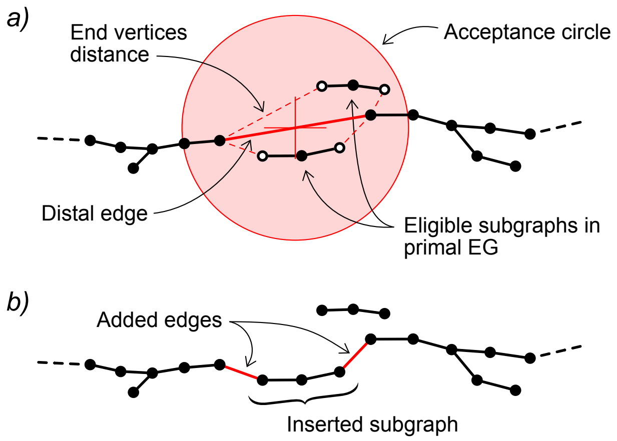

Figure S3. Recovery of small subgraphs corresponding to EG long edges. Subgraphs in primal EG are eligible for distal edge replacement if at least two end vertices (empty dots) are positioned inside the acceptance circle (a). The final subgraph to be inserted (if any) is the one with the smallest distances between its end vertices and the distal edge vertices (see text for details). The selected subgraph is inserted in the crack EG by replacing the former distal edge with two new edges (b).

S3. Artefact removal algorithm

In this work, an algorithm is used for the removal of skeletonization artefacts based on the identification, on each crack, of the main crack path and its lateral branches. The algorithm is implemented by a series of shortest path searches. The polished crack EG is then built by assembling the located main path with the lateral branches in decreasing size order identified thereafter. The main advantages of this approach are the elimination of every residual cycle in the EG (that, given the specific samples investigated in this work, are all assumed to be artefacts) and the identification of all significant crack branches with contextual removal of small branches and parasitic features generated in the skeletonization process (pruning).

The main crack path of each crack subgraph (Figure S4a) is located by finding the largest of all the shortest paths connecting two end-vertices of the subgraph itself (Figure S4b). The first (largest) secondary branch is identified by evaluating all the shortest paths that depart from the remaining end vertices and reach the main path, then selecting the largest one (Figure S4c). Further branches can be identified by iterating the latter procedure with the remaining unassigned end vertices (Figure S4d–f). The procedure is terminated when the obtained branch size is lower than a given minimum limit

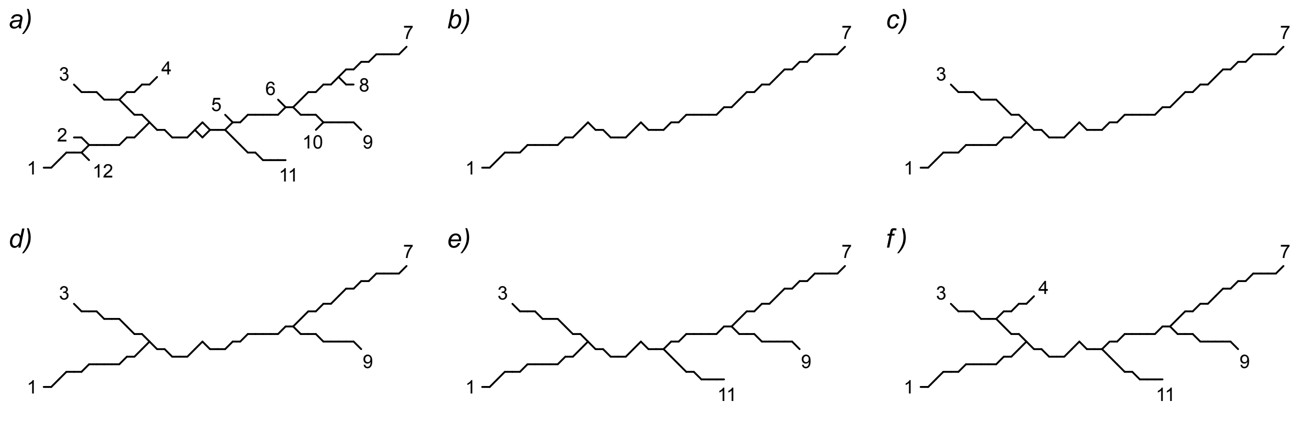

Figure S4. Artefact removal algorithm. A crack EG with 12 end vertices shows several skeletonization artefacts as parasitic small arms and cycles (a). The main path of the crack is located on the EG seeking the most distant end vertices and computing the shortest path between them (b). Secondary branches are found seeking shortest paths from the remaining end vertices to the already found path in decreasing size order until the minimum limit is reached, effectively removing all artefacts (c–f).

or when no more unassigned end vertices are available on the crack subgraph. The sub-EG obtained by adding all found paths (Figure S4f) is therefore free from secondary branches smaller than the minimum allowable size limit and it is also free from cycles (Section S3.1). Moreover, the described algorithm allows to build a hierarchy of lateral branches ordered by size that can also be classified on the basis of their branch connection (*i.e.* as secondary branch if connected to the main path, as tertiary branch if connected to a secondary branch, and so on). In this study, the size of the main and secondary branches is defined as the path length (calculated as sum of all edge lengths). All paths are then located by the Dijkstra's shortest path search algorithm (Section S3.1). However, EG-based crack description allows to use other path size definitions (*e.g.* the path vertices count), the final choice depending mainly on analysis goals and computational cost. Moreover, the use of EG path descriptions allows to implement an effective optimization transforming the crack EG into an equivalent, more compact representation based on weighted graphs (Section S3.2).

S3.1 Shortest path search algorithm

The main crack and the lateral branch paths are computed with the Dijkstra's algorithm for shortest path search in weighted graphs, where the edge weights are set according to the Euclidean distances between its vertices. The main path is located on the crack subgraph looking for the longest of all shortest paths in the crack EG connecting two end-vertices. This can be carried out by running the Dijkstra's algorithm from each end vertex (and also from vertices with degree ≥3 in case that some branch ends in a small cycle instead of a degree-1 vertex). The shortest path from an end vertex to the located main path can then be evaluated by running the Dijkstra's algorithm from the end vertex itself until it reaches a vertex belonging to the main path. The search for the first lateral branch is performed by running this latter procedure from all the remaining end vertices and selecting the longest path found. The located lateral branch is then added to the main path in the polished EG. All the remaining branches can be later located in decreasing length order by iterating the search process and adding each found path to the EG under construction. The process is terminated when there are no more unassigned end vertices available or the last obtained lateral branch length is lower than the given minimum limit. If a branch classification based on the branch degree is needed (*i.e.* first order if main path, second order if branch attached to the main path, third order if branch attached to a first order branch and so on) this may be easily accomplished by labeling each vertex, as soon as it is located, with its respective degree. Notice that no cycles can be contained in the polished EG because it is built starting from the main path (that is cycle-free because is the result of a shortest path search) and adding all the successive secondary branches (that are also cycle-free because they are all shortest paths starting from end vertices).

S3.2 Search with smaller weighted graph

The localization of the crack main path and the lateral branches on the EG can be a computationally intensive task because large crack subgraphs can have dozens or more end vertices. In the present context, however, all searches start from and end in branching or end vertices. An internal path of degree 2 (*i.e.* constituted exclusively by vertices with degree 2) is therefore always traversed by the search algorithm that never ends on any of its vertices. It is thus possible to build a simplified

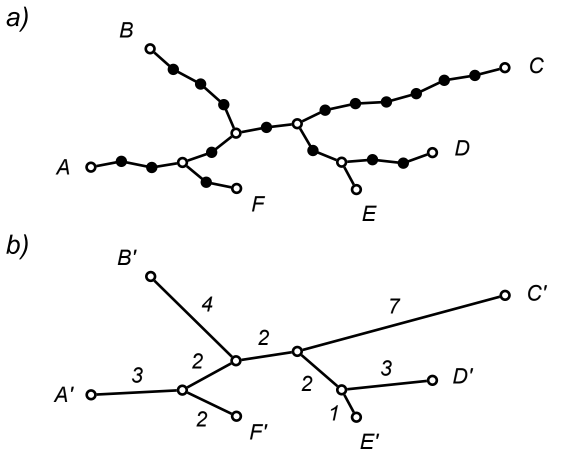

Figure S5. Compact weighted graph for shortest path search in crack EGs. A crack EG (a), here with all unitary length edges, can be replaced by an equivalent weighted graph (b) where each internal path composed by degree 2 vertices (filled dots) is replaced by a single edge with weight equal to the sum of all of its components. All vertices with degree other than 2 are preserved (empty dots). For each pair of corresponding paths in (a) and (b) the length of the former is equivalent to the path weight of the latter (*e.g.* the path between the end vertices *A′–C′* in the weighted graph has a cumulative weight equal to the length of the corresponding path *A–C* in the EG).

weighted graph replacing each of these sub-paths with a single edge, provided that its weight is the sum of all the lengths of the replacing edges (Figure S5). All other edges in the weighted graph are assigned a weight equivalent to the edge length in the original EG. If two internal paths end in the same pair of vertices, the weight corresponding to the shortest one is applied to the single replacing edge (in more general contexts, special cases like the latter must be however handled specifically, see Figure S6).

The resulting compact weighted graph is equivalent, from the search algorithm standpoint, to the original EG. This conversion can be particularly valuable for crack EGs because they consist mostly of quite long chains of degree-2 vertices, thus resulting in a remarkable size reduction and, consequently, in a shorter search time. After performing the search on the compact graph, the complete path can be located on the full crack EG by evaluating on it only the shortest path between the two found end vertices.

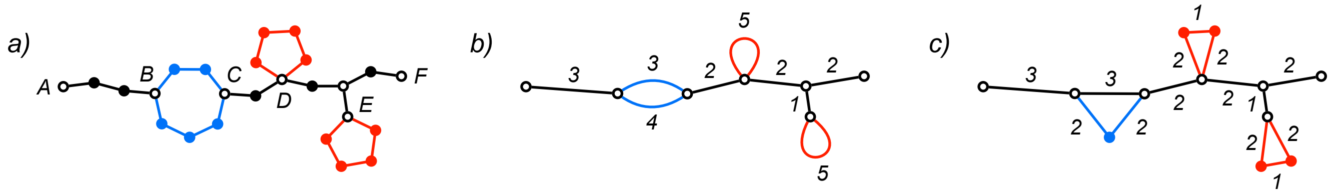

Figure S6. Special cases in compact weighted graph generation from a generic EG (a), such as multiple internal paths ending in the same vertex pair (blue) or cycles starting and ending in the same vertex (red), can be handled with a weighted multigraph representation (b) or by partially retaining the selected degree-2 vertices (filled dots). All edges in the depicted EG have unitary length (a); numbers indicate edge weight (b, c).

S4. Crack correlation search algorithm

Possible correlations in contiguous cracks are seeked by using a specific algorithm based on the relative orientation and distance of their respective EG end parts (Figure S7).

Two cracks are considered part of a bigger crack if both eligible end vertices fall within an acceptance region (Figure S7b) computed from the complementary end vertex and if the relative angle of the crack end directions falls within a given acceptance window (Figure S7c). Eligible cracks are quickly located with a preliminary proximity screening based on rectangles encompassing all end vertices positions of each crack EG representation. All end vertices of each eligible crack pair are then evaluated against all end vertices of the complementary crack. The crack end direction for the evaluation of the acceptance region and the relative angle is calculated with the linear fitting of vertices near the end vertex, which can be easily obtained from the crack EG.

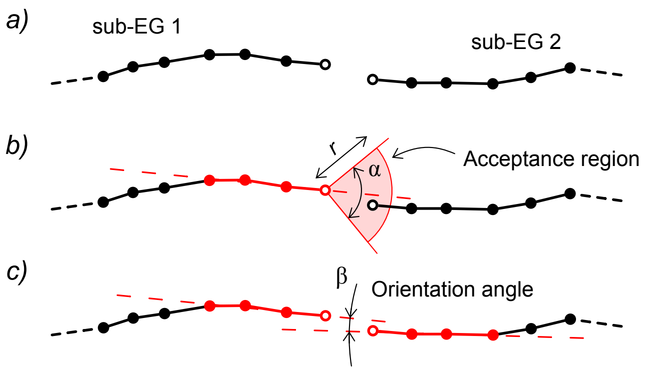

Figure S7. Contiguous crack correlation. Two cracks (a) are considered parts of a bigger crack if the end vertices of the two crack sub-EGs fall within the complementary end vertex acceptance region (b) and the relative angle of the terminal parts (c) is within an acceptance range. Direction of crack end is computed with terminal path vertices (in red).

S5. EG–EG quantitative comparison algorithm

There are many reasons to compare different EGs, including the quality evaluation of an acquisition process (by correlating the resulting EG with a reference EG using suitable performance metrics) and the assessment of crack time evolution (by comparing EGs of the same crack acquired at different times). Correlation may be established between two crack EGs by determining the length of their respective matching path (*i.e.* the length of the path parts of each EG that overlaps with the other, assuming that the spatial position of both EGs vertices belongs to the same coordinate system). The overlapping path is defined as the subpath of each EG that whose distance from the path of the other EG is not greater than a given tolerance (Figure S8). An overlap area can thus be established for each EG within which a second EG can be defined as overlapping (Figure S9a). It is worth noting that with this definition some edges can be considered both partially overlapping and non-overlapping. If the maximum distance between each pair of connected vertices (*i.e.* the maximum length for all EG edges) is significantly smaller than the overlap tolerance, the overlap area can be approximated by the union of points that are no farther than the tolerance distance from at least one EG vertex. In this situation the EG–EG correlation can be carried out (with reasonable approximation) by direct comparison of the vertex positions of each EG. If, however, there are edges with length comparable to or greater than the overlap tolerance, these must be replaced by a series of collinear connected nodes in order to fulfill the above condition (Figures S9b–S9c). In this work, the maximum allowable edge length is set to half the overlap tolerance and each longer edge is subdivided into several shorter ones. Therefore, assuming the aforementioned conditions, in order to compare two crack EGs (EG_1 and EG_2) it is possible to use the following procedure, which must be iterated for each vertex V in EG_1:

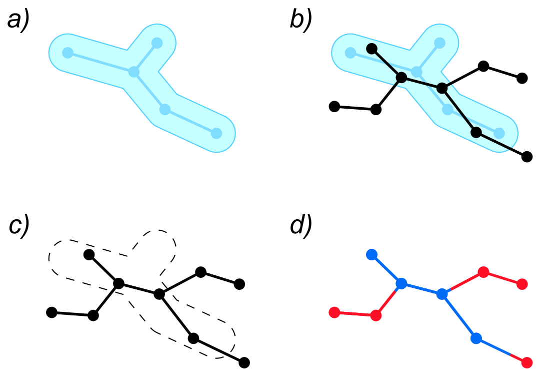

Figure S8. EG-EG path comparison. A reference EG with a defined overlap tolerance area (a). A sample EG (black) superimposed to the reference EG (b). The part of the sample EG corresponding to the reference EG falls within the overlap area while the non-matching part falls outside it (c). Sample EG with path labeled as matching (blue) or non-matching (red) with some edges partially attributed to both categories.

1) The addition of the length of all edges connected to V is computed.
2) A vertex V' is searched in EG_2 with V–V' distance less than or equal to the overlap tolerance.
3) If the vertex V' is found, the vertex V on EG_1 is labeled as overlapping and the half of the computed edge length sum is added to the overlap accumulator.
4) If the vertex V' is not found, the vertex V on EG_1 is labeled as non-overlapping and the half of the computed edge length sum is added to the non-overlap accumulator.

At the end of all iterations, the overlap accumulator contains the estimated cumulative length of the parts of EG_1 path that overlap with EG_2, while the non-overlap accumulator contains the estimated length of the remaining EG_1 path parts non-overlapping with EG_2. Moreover, all the vertices in EG_1 are identified either as overlapping or as non-overlapping with EG_2. The procedure is then repeated by inverting the EGs in order to find the parts of EG_2 that overlaps (or does not overlap) with EG_1. It is important to notice that the overlapping parts of EG_1 and EG_2 can have (slightly) different length because they are obtained from different EGs. In order to perform a quality evaluation of a sample crack EG (EG_S) using

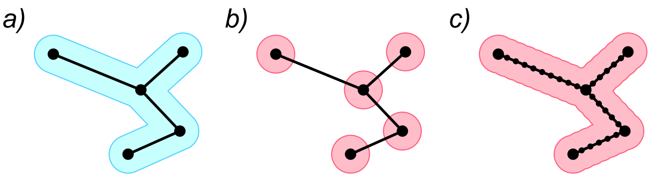

Figure S9. EG overlap area (blue) defined for a given tolerance (a). The union of the circles centered on the EG nodes (red) with radius equal to the overlap tolerance can effectively approximate the overlap area if the maximum edge length is not longer than a fraction of the overlap tolerance. An EG with longer edges (b) can be replaced with an equivalent EG (c) with additional nodes (small dots) in order to ensure the correct maximum edge length (here depicted as half of the overlap tolerance).

a reference ground truth EG (EG_R), it is possible to define the following quantities:

$$TP = (O_S + O_R)/2 \quad (1)$$

$$FP = N_S \quad (2)$$

$$FN = N_R \quad (3)$$

where O_S is the length of the EG_S path that overlaps with EG_R, N_S is the length of the EG_S path that does not overlap with EG_R, O_R is the length of the EG_R path that overlaps with EG_S, N_R is the length of the EG_R path that does not overlap with EG_S, TP is the true positive (*i.e.* the length of the path correctly identified by EG_S), FP is the false positive (*i.e.* the length of EG_S path not corresponding to EG_R) and FN is the false negative (*i.e.* the length of EG_R path that is not identified by EG_S). Here the TP value is calculated as the mean of the sample and the reference overlapping paths.
With the TP, FP and FN defined above, several well-known quality evaluation indicators can be computed based on the matching EG path length instead of the typical segmentation pixel count, including the following:

$$P = \frac{TP}{TP + FP} \quad (4)$$

$$R = \frac{TP}{TP + FN} \quad (5)$$

$$IoU = \frac{TP}{TP + FP + FN} \quad (6)$$

$$F1 = 2 \cdot \frac{P \cdot R}{P + R} \quad (7)$$

where P is the precision, R is the recall (also known as sensitivity), IoU is the intersection over union and F1 is the F1-score (all dimensionless).

S6. EG smoothing algorithm

In this work, a simple iterative algorithm for EG smoothing is implemented that acts on internal (degree 2) vertices, leaving untouched all end vertices (degree 1) and all branching vertices (degree ≥3). For each iteration, every internal vertex position is replaced by a new vertex placed in the midpoint between the vertex position itself and the preceding one on the EG path. Referring to the example given in Figure S10, the internal vertex *2* (Figure S10a) is replaced (Figure S10b) by a new vertex (*2′*, red) positioned in the midpoint between vertex *2* and the preceding end vertex *1* (that is left untouched). Vertex *3* is replaced by a new vertex (*3′*) placed in the midpoint between vertices *2* and *3*, and so on for all the remaining internal vertices. A new vertex is then inserted in the midpoint between the last internal vertex and the connected branching or end vertex (*6′*). This process results in the first smoothing iteration cycle, where all internal vertices are replaced and the total EG vertex count is incremented by one (Figure S10c). The same mechanism is applied to the second cycle (Figure S10d), resulting in a new EG with one more vertex (Figure S10e). The optimal number of cycles is determined by the final application. The described procedure results in a new EG with all branching and end vertices unmodified but all the existing internal vertices are moved to new positions (and with some new internal vertices added).

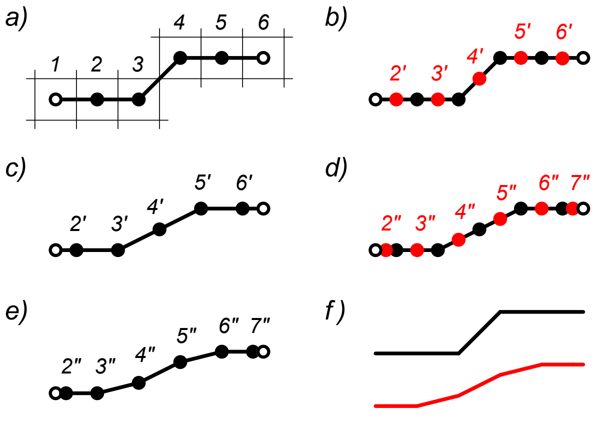

Figure S10. EG smoothing algorithm. Internal vertices in initial EG (a) are replaced by new vertices (red dots) located in the midpoint of each edge (b). The resulting smoothed EG with one more internal vertex (c) is subjected to a new iteration (d) giving a two-cycle smoothed EG (e). In (f) the pristine EG (black) and the 2-cycles smoothed EG (red) are compared.

S7. Crack edge identification algorithm

The local crack width can be defined as the distance of the intersection points between the crack edges from a given measurement line. The latter can be either perpendicular to the crack tangent in the width measurement point or, as in the present work (Figure S11a), parallel to a given fixed direction (the strain vector). The crack appears as a valley on the image intensity profile computed along the measurement line (Figure S11b). The crack edges can thus be found by locating the first intersection (starting from the centre of the crack) of the image intensity profile with an appropriate threshold value (Figure S11c).
The determination of the best threshold value for crack edge estimation is thus a critical step for width measurement. In the present study, the threshold value is computed locally for each measurement line with the algorithm described below, aimed

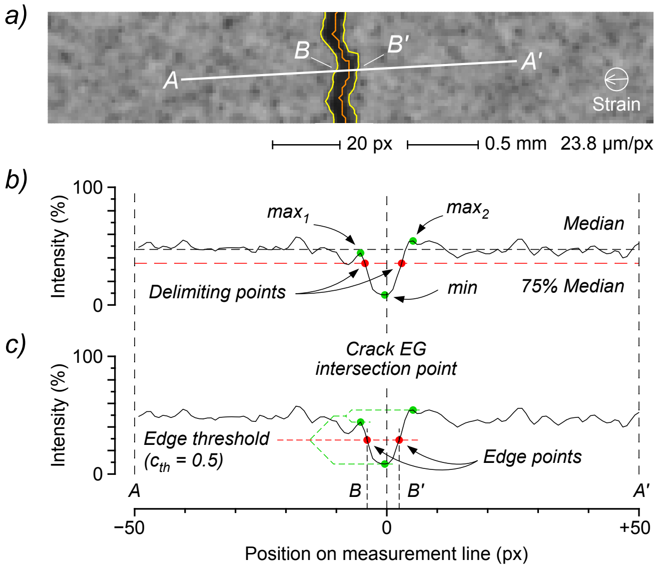

Figure S11. Crack width determination. Crack image (a) with a measurement line (*A–A′*, white), a crack EG (orange) and two detected crack edges (yellow). Intensity profile along the measurement line (b) with identified points for edge threshold determination (*min*, max_1 and max_2). Intensity profile (c) with crack edge threshold and crack edge point determination (*B* and *B′*). The measurement line depicted here is very short for the sake of clarity (typical sampling point count is >200).

at finding the relevant intensity values for both the deep crack and the sample surface even in the presence of significant surface texture. Firstly, a measurement line is established with the center located on the EG path. Two boundary points are then identified on the measurement line departing from its central point (that is located inside the crack because it lies on the intersection with the crack EG) and moving in both directions until the sampled image intensity exceeds a specific threshold value. The latter is defined as 75% of the median intensity value of all sampled points (Figure S11b). Two maximum intensity values max_1 and max_2, outside the boundary points, are then located moving outwards until a local intensity maximum is found (*i.e.* until the last value of monotonic intensity increase). The minimum value *min* is calculated as the minimum intensity value of sampled points between the identified boundary points. The edge threshold value *th* is finally calculated as the linear combination between the minimum and the mean of the two maximum intensity values found, as in the following:

$$th = (1 - c_{th})\ min + c_{th} \frac{max_1 + max_2}{2} \qquad 0 \le c_{th} \le 1 \qquad (8)$$

where c_{th} is the linear combination coefficient. The value for c_{th} in this study is 0.5 (*i.e.* at the midpoint between the minimum and the mean of the maximum values). The two crack edges are finally located by finding the first intersection of the sampled intensity profile with *th*, departing from the measurement centre of the line (Figure S11c). Thanks to this procedure, no local maximum (value) is captured in the depth of a large crack, where image noise can easily produce spurious fluctuations. A distinct advantage is the possibility, when required, to adjust the coefficient c_{th} with a specific calibration in order to improve the final measurement precision. Width values obtained along the crack path can be used to calculate the averaged crack width or to build the crack width profile. Because the measurement line is arbitrary-oriented, the related image intensity profile is determined by calculating a series of sampling points along the measurement line itself by interpolating the neighboring image pixel values. The method used in this work is the bilinear interpolation of the four pixels nearest to each sampling point.
